# Self-reported mental health during the COVID-19 pandemic and its association with alcohol and cannabis use: a latent class analysis

**DOI:** 10.1186/s12888-022-03917-z

**Published:** 2022-04-30

**Authors:** Nibene Habib Somé, Samantha Wells, Daniel Felsky, Hayley A. Hamilton, Shehzad Ali, Tara Elton-Marshall, Jürgen Rehm

**Affiliations:** 1 Institute for Mental Health Policy Research, Centre for Addiction and Mental Health, 100 Collip Circle, Suite 200, ON N6G 4X8 London, Canada; 2 Campbell Family Mental Health Research Institute, Centre for Addiction and Mental Health, Toronto, Ontario Canada; 3 Institute for Clinical Evaluative Sciences, Toronto, Ontario Canada; 4 Department of Epidemiology and Biostatistics, Schulich School of Medicine and Dentistry, Western University, London, Ontario Canada; 5 Dalla Lana School of Public Health, University of Toronto, Toronto, Ontario Canada; 6 Department of Psychiatry, University of Toronto, Toronto, Ontario Canada; 7 School of Psychology, Deakin University, Victoria, Australia; 8 Institute of Medical Science, University of Toronto, Toronto, Ontario Canada; 9 Krembil Centre for Neuroinformatics, Centre for Addiction and Mental Health, Toronto, Ontario Canada; 10 Schulich Interfaculty Program in Public Health, Schulich School of Medicine and Dentistry, Western University, London, Canada; 11 Department of Health Sciences, University of York, York, UK; 12 Department of Psychology, Macquarie University, Sydney, Australia; 13 Bruyere Research Institute, Ottawa, Canada; 14 School of Epidemiology and Public Health, Faculty of Medicine, University of Ottawa, Ottawa, Canada; 15 Department of Health Sciences, Lakehead University, Thunder Bay, Ontario Canada; 16 Institute for Clinical Psychology and Psychotherapy, TU Dresden, Dresden, Germany

**Keywords:** COVID-19, Latent class analysis, Mental health, Alcohol, cannabis

## Abstract

**Background:**

Mental health problems and substance use co-morbidities during and after the COVID-19 pandemic are a public health priority. Identifying individuals at high-risk of developing mental health problems and potential sequela can inform mitigating strategies. We aimed to identify distinct groups of individuals (i.e., latent classes) based on patterns of self-reported mental health symptoms and investigate their associations with alcohol and cannabis use.

**Methods:**

We used data from six successive waves of a web-based cross-sectional survey of adults aged 18 years and older living in Canada (6,021 participants). We applied latent class analysis to three domains of self-reported mental health most likely linked to effects of the pandemic: anxiety, depression, and loneliness. Logistic regression was used to characterize latent class membership, estimate the association of class membership with alcohol and cannabis use, and perform sex-based analyses.

**Results:**

We identified two distinct classes: (1) individuals with low scores on all three mental health indicators (no/low-symptoms) and (2) those reporting high scores across the three measures (high-symptoms). Between 73.9 and 77.1% of participants were in the no/low-symptoms class and 22.9–26.1% of participants were in the high-symptom class. We consistently found across all six waves that individuals at greater risk of being in the high-symptom class were more likely to report worrying about getting COVID-19 with adjusted odds ratios (aORs) between 1.72 (95%CI:1.17–2.51) and 3.51 (95%CI:2.20–5.60). Those aged 60 + were less likely to be in this group with aORs (95%CI) between 0.26 (0.15–0.44) and 0.48 (0.29–0.77) across waves. We also found some factors associated with class membership varied at different time points. Individuals in the high-symptom class were more likely to use cannabis at least once a week (aOR = 2.28, 95%CI:1.92–2.70), drink alcohol heavily (aOR = 1.71, 95%CI:1.49–1.96); and increase the use of cannabis (aOR = 3.50, 95%CI:2.80–4.37) and alcohol (aOR = 2.37, 95%CI:2.06–2.74) during the pandemic. Women in the high-symptom class had lower odds of drinking more alcohol during the pandemic than men.

**Conclusions:**

We identified the determinants of experiencing high anxiety, depression, and loneliness symptoms and found a significant association with alcohol and cannabis consumption. This suggests that initiatives and supports are needed to address mental health and substance use multi-morbidities.

**Supplementary information:**

The online version contains supplementary material available at 10.1186/s12888-022-03917-z.

## Introduction

In Canada, depression and anxiety disorders are among the most common mental health disorders and have been shown to have a major impact on the daily lives of those affected [1, 2]. Three million Canadians (11.6%) aged 18 years or older reported having a depression and/or anxiety disorder in 2013 [3]. Evidence indicates that the COVID-19 pandemic and related public health directives (e.g., lockdowns) have led to elevated mental health symptoms, including depression, anxiety and loneliness among individuals worldwide [4–10]. As such, a better understanding of the effect of the pandemic on people’s mental health as well as associated substance use (i.e., alcohol drinking and cannabis use) is needed to inform public health interventions.

The first case of COVID-19 in Canada was reported in Ontario on January 25, 2020 [11]. As of December 2021, there had been about 1.8 million positive COVID-19 cases and over 29,000 deaths reported in Canada [12]. Public health measures were implemented across Canada at the provincial level with different timing and intensity, including stay-at-home orders, bans on large public gatherings, physical distancing, self-isolation, and quarantines. Although these measures were successful in slowing the spread of the virus in jurisdictions across the country, evidence suggests they had negative effects on people’s mental health and well-being [13, 14].

During the pandemic, about 19% of adult Canadians screened positive for either symptoms of anxiety and/or depression [15]. By comparison, only 8.9% of Canadians reported mental health symptoms prior to the pandemic (i.e., from October to December, 2019) [16]. A similar increase in mental health problems has been found among adults in the US [17]. Similarly, although some studies in the UK and Italy have shown that people have developed psychological resilience during the pandemic [18–22], numerous studies indicate that the pandemic and related public health directives have increased mental health symptoms among individuals worldwide [4–10]. In a review of 19 studies of the general population, higher scores of anxiety and depression were found compared to before the pandemic [23]. Additionally, several studies have shown that stay-at-home orders, lockdowns and physical distancing have increased loneliness [24–26], which in turn is linked to both depression and anxiety [27–30]. Though important for reporting prevalence rates, these studies lack data on patterns across mental health conditions. With the rise in depression and anxiety and their frequent co-occurrence [31–33], as well as their link with loneliness [27–30], it is important to simultaneously examine these three mental health indicators to unveil patterns in their co-occurrence.

Elevated depression, anxiety and loneliness during the pandemic may be linked to adverse health behaviors, including substance use [34]. Thus, it is important to investigate whether patterns in co-occurring mental health symptoms are associated with substance use during the pandemic. Recent research has shown that individuals are consuming more alcohol [35–38] and more cannabis [35, 39, 40] than they did before the pandemic. Such patterns in substance use may result in acute and chronic harms, such as injury, substance use dependence, and death [41–44]. People may be using more alcohol and cannabis to cope with anxiety, depression and loneliness experienced during the pandemic [45–47]. Moreover, people experiencing co-occurring mental health symptoms (e.g., anxiety, depression and loneliness) may be especially likely to use substances during the pandemic [48, 49].

Sex-differences in psychological distress have been shown in the literature before the pandemic [50–52]. Studies during the pandemic have found that, compared to men, women reported more problems regarding mental health issues (e.g., depression and anxiety) [38, 53–55]. In Canada, prior to the pandemic, women were more likely than men to report fair/poor mental health (8.6% vs. 6.7%) [56], with the pandemic these proportions have increased to 25.5% for women and 21.2% for men [56]. Additionally, some research suggests that women (compared to men) are more likely to drink alcohol to cope with psychological distress [57, 58], while other studies reported significant associations between increased emotional distress and increased alcohol and cannabis use during the pandemic for both men and women [59, 60], and only among men [61]. Thus, it is important to investigate sex-differences in this study.

The present study aims to identify distinct groups of individuals (i.e., latent classes) based on patterns of self-reported mental health symptoms and examine their associations with substance use. The specific objectives are to:

1. Identify different latent classes of mental health symptoms and examine factors associated with class membership, including socio-demographics and worry about contracting COVID-19, and whether these associations differ by sex or change over time;
2. Assess the associations between the mental health latent classes and individuals’ alcohol and cannabis use during the pandemic, and whether associations differ by sex and over time.

These objectives are achieved using repeat cross-sectional surveys conducted in Canada and latent class analysis (LCA), a statistical method that creates groups of individuals with similar patterns of characteristics referred to as latent classes [62]. LCA is recognized as a useful tool for studying and classifying mental health disorders at the population level [63]. Since positive associations have been found among depression, anxiety, and loneliness, we expect to find a distinct class of individuals with a high probability of reporting co-occurring mental health symptoms (i.e., depression, anxiety, and loneliness). Understanding such patterns is important because mental health multi-morbidities are associated with reduced quality of life [64, 65], are more difficult to treat and may be differentially associated with substance use challenges [66, 67]. Moreover, the identification of groups of individuals with mental health multi-morbidities has important implications for public health policy, including resource allocation, raising awareness, and appropriate screening. In addition, it may inform the design of interventions or tailoring of existing interventions to meet the needs of people with multi-morbidities, particularly when they are at risk of elevated substance use.

## Methods

### Study design and participants

This study used data from six successive waves of web-based cross-sectional Canada-wide surveys of adults aged 18 years and older. The surveys were conducted in English by the firm Delvinia. The sample was derived from a web-based survey panel, and quota sampling was used to approximate the distribution of the English-speaking Canadian population by age, sex, and region [68]. Electronic informed consent was obtained before initiating the survey. The study received ethics approval from the Centre for Addiction and Mental Health. The surveys were conducted in six waves in 2020 as follows: May 8–12 (Wave 1, *n* = 1,005, response rate (RR) = 15.9%), May 29-June 1 (Wave 2, *n* = 1,002, RR = 17.2%), June 19–23 (Wave 3, *n* = 1,005, RR = 16.4%), July 10–14 (Wave 4, *n* = 1,003, RR = 13.7%), September 18–22 (Wave 5, *n* = 1,003, RR = 17.6%), and November 27-December 1 (Wave 6, *n* = 1,003, RR = 16.2%). The details of the survey interviews information and RR calculations are in Table A.1 of the Additional file 2. A pooled sample of 6,021 participants (Waves 1–6) was analyzed in this study. These data were collected at different points to permit an examination of variation in the impact of COVID-19-related stressors on participants over time.

### Measures

#### Mental health indicators

We identified anxiety among participants using the 7-item generalized anxiety disorder, using the GAD-7 scale based on 4-point Likert-scale questions. These items measure the frequency of anxiety symptoms over the past two weeks and are scored from 0 (not at all) to 3 (nearly every day). The summary score ranged from 0 to 21 [69]. A score $\ge$10 suggests moderate or severe anxiety to consider treatment [70] which has clinical relevance. The literature that has studied GAD-7 scale has also validated the cut-off of 10 [70–72]. We then constructed a binary variable for anxiety to identify participants with moderate or severe anxiety symptoms [73].

Participants who felt depressed were identified using a question from the Center for Epidemiologic Studies Depression Scale (CES-D) [74]: “In the past 7 days, how often have you felt depressed?” Response options included: “rarely or none of the time (less than 1 day)”, “some or a little of the time (1–2 days)”, “occasionally or a moderate amount of the time (3–4 days)”, and “most or all of the time (5–7 days)”. Participants who reported feeling depressed 3–4 or more days in the previous week were classified as experiencing depressive symptoms [74]. Similarly, loneliness was measured with a single item from the CES-D [74] with the same response options: “In the past 7 days, how often have you felt lonely?” Participants were considered to be lonely if they reported feeling lonely for 3–4 or more days in the previous week [74].

Although LCA is a data-driven method, extra steps are needed to ensure that identified classes are interpretable and not simply statistical artefacts [75]. We described the classes and determined the factors that are associated with the classes.

#### Alcohol and cannabis use variables

Four variables related to alcohol and cannabis use were assessed. For alcohol, a binary variable identifying heavy episodic drinkers was derived based on the responses to the question: “On how many of the past seven days did you drink four (if a woman) or five (if a man) or more drinks on one occasion?” Men who consumed five (four for women) or more drinks per occasion at least four days per week were coded as heavy episodic drinkers. Note that a drink was defined as a 12 oz. bottle of beer or cider/cooler (5% alcohol content), a 5 oz. glass of wine (12% alcohol content), or a straight or mixed drink with 1.5 oz. of liquor (40% alcohol content). The second alcohol use question examined whether people’s drinking increased due to the pandemic. Participants were asked: “In the past seven days, did you drink more alcohol, about the same or less alcohol overall than you did before the COVID-19 pandemic started?” This measure was coded to reflect an increase in alcohol use as: 0 (much less, slightly less, or same), and 1 (slightly more or much more).

For cannabis use, participants were asked: “During the past seven days, on how many days did you use cannabis?” A binary measure was created to reflect any cannabis use (use on one or more days) versus no cannabis use in the past week. Increase in cannabis use was also measured with the question: “In the past 7 days, did you use cannabis more often, about the same, or less often overall than you did before the COVID-19 pandemic started?” This was coded to reflect an increase in cannabis use as follows: 0 (much less, slightly less, or same), and 1 (slightly more or much more).

#### Covariates

We included several individual and household covariates: sex, age (18–39, 40–59 and 60 years or more), marital status (married/living with a partner, separated/divorced/widowed and single), educational status (high school or less, some post-secondary, college degree/diploma and university degree/diploma), racial group (White and non-White, i.e., Asian, Black/Indigenous/Arab/Latinos and other ethnicities), residential environment (urban, suburban and rural), household income (less than $40,000, $40,000-$79,999, $80,000-$119,999, $120,000 or more, and ‘prefer not to answer’), having children under 18 in the household and household composition (living alone or living with others). We also included a variable indicating the extent of worry experienced regarding contracting COVID-19, based on responses to the question: “How worried are you that you or someone close to you will get ill from COVID-19?”, with possible responses provided on a 4-point Likert scale of: “very worried,” “somewhat worried,” “not very worried,” and “not at all worried.” We derived a binary variable to compare those classified as worried (i.e., very or somewhat worried) versus those not worried (i.e., not very or not at all worried). We accounted for time effects by adding a binary variable for each wave.

### Statistical analyses

We used LCA to identify classes of participants with similar patterns of reported mental health symptoms during the pandemic. We used the three mental health indicators (anxiety, depression, loneliness) to divide participants into mutually exclusive and exhaustive latent classes. Using LCA, we estimated the probability for each participant of being in a particular class based on their responses to all three indicator items. We used the gsem command in Stata and specified logit to fit logistic regression models for all three indicators. We estimated intercept-only models for each indicator by selecting the number of latent classes. To determine the optimal number of latent classes, we estimated latent class models using different class numbers, and we used Akaike’s (AIC) and the Bayesian information criterion (BIC) to select the model with the better fit [76]. We used logistic regression to determine risk factors associated with latent classes membership.

To assess the associations of latent class memberships with alcohol and cannabis use, we used multivariate logistic regression. We adjusted for individual participant confounders (sex, age, education, marital status, ethnicity, residential environment), household confounders (income, presence of children, presence of other persons in the home), worrying about getting COVID-19, and survey wave indicator variables. We tested for sex differences by including latent class by sex interactions. We also included latent class by wave interaction terms to assess whether and how the association of class membership with alcohol and cannabis use changed over time. We then calculated the F-test for the joint significance of interaction terms to detect time/wave effects.

We presented descriptive statistics of the cohort, including percentages and number of observations. We also reported adjusted odds ratios (aORs) with 95% confidence intervals (CIs), and presented results by sex. We used Stata (version 16.0) for all analyses. The full estimation tables are in the Additional file 2 (Table A.2–3).

## Results

A total of 6,021 participants completed the survey across the six waves, with at least 1,000 participants per wave. In Table 1, we report the number and the percentage of participants for each self-reported measure of mental health symptom, alcohol and cannabis use, participants’ characteristics within each wave, and for the total sample (all six waves). Overall, the percentage of participants who reported severe/moderate anxiety, depression, and loneliness were quite similar across the waves and in the entire sample (ranging between 19 and 25%, 18–22%, and 20–24%, respectively). Between 12 and 16% of participants reported using cannabis at least once a week, and 24–27% reported engaging in heavy episodic drinking. Regarding change in cannabis and alcohol use, a total of 401 (7%) and 1,295 (22%) participants reported having increased their use of cannabis and alcohol.

### Latent class modeling and identification of classes

Models with one to four latent classes for each wave and pooled waves were estimated and compared using the information criteria (see Table A.4 in the Additional file 2). All three criteria (Log-likelihood, AIC, and BIC) indicated that the two-class models fit better than other models. However, we also characterized the three-class model and estimated the association between the three-latent class variable and substance use to assess the sensitivity of the results to the number of classes (results for the three-class model are in Table A.5–6 in Additional file 2).

**Table 1:** Descriptive statistics: Mental health indicators, substance use, and sociodemographic characteristics

	Wave 1		Wave 2		Wave 3		Wave 4		Wave 5		Wave 6		All waves	
Variables	n	%	n	%	n	%	n	%	n	%	n	%	n	%
*Mental health indicators*														
Moderate/severe anxiety	256	25.5%	215	21.5%	196	19.5%	193	19.2%	212	21.1%	244	24.3%	1319	21.9%
Felt depressed	205	20.4%	212	21.2%	185	18.4%	188	18.7%	213	21.2%	218	21.7%	1222	20.3%
Felt lonely	233	23.2%	237	23.7%	211	21.0%	231	23.0%	202	20.1%	234	23.3%	1349	22.4%
*Alcohol and cannabis use*														
Cannabis use past week	115	11.5%	130	13.0%	124	12.4%	131	13.1%	119	11.9%	160	16.0%	781	13.0%
Heavy Episodic Drinking	238	23.7%	247	24.7%	267	26.6%	271	27.2%	255	25.5%	257	25.7%	1537	25.6%
Increase in cannabis use	64	6.4%	70	7.0%	59	5.9%	62	6.2%	53	5.3%	93	9.3%	401	6.7%
Increase in alcohol use	253	25.2%	244	24.4%	216	21.5%	209	20.8%	168	16.7%	208	20.7%	1295	21.5%
*Covariates*														
Men	504	50.1%	492	49.1%	501	49.9%	502	50.0%	497	49.6%	492	49.1%	2986	49.6%
Women	498	49.6%	497	49.6%	499	49.7%	492	49.1%	498	49.7%	503	50.1%	2986	49.6%
Living with others	797	79.5%	788	78.9%	787	78.7%	799	79.9%	797	79.6%	796	79.8%	4763	79.4%
Living alone	205	20.5%	211	21.1%	213	21.3%	201	20.1%	204	20.4%	201	20.2%	1236	20.6%
Presence of children	229	22.8%	236	23.6%	237	23.6%	242	24.1%	234	23.3%	216	21.5%	1397	23.2%
No children	776	77.2%	766	76.4%	768	76.4%	761	75.9%	769	76.7%	787	78.5%	4624	76.8%
Household income less than $40K	128	12.7%	121	12.1%	136	13.5%	118	11.8%	116	11.6%	110	11.0%	729	12.1%
Household income in $40,000-$79,999	268	26.7%	236	23.6%	238	23.7%	235	23.4%	247	24.6%	236	23.5%	1457	24.2%
Household income in $80,000-$119,999	226	22.5%	229	22.9%	220	21.9%	213	21.2%	237	23.6%	241	24.0%	1367	22.7%
Household income $120,000+	217	21.6%	259	25.8%	247	24.6%	252	25.1%	228	22.7%	251	25.0%	1451	24.1%
Household income missing	166	16.5%	157	15.7%	164	16.3%	185	18.4%	175	17.4%	165	16.5%	1012	16.8%
College	190	18.9%	211	21.1%	189	18.8%	204	20.3%	183	18.2%	221	22.0%	1198	19.9%
High school	111	11.0%	104	10.4%	129	12.8%	122	12.2%	119	11.9%	99	9.9%	686	11.4%
Post-secondary	159	15.8%	165	16.5%	148	14.7%	162	16.2%	147	14.7%	150	15.0%	933	15.5%
University	538	53.5%	516	51.5%	531	52.8%	502	50.0%	548	54.6%	521	51.9%	3155	52.4%
Non-White	287	28.6%	271	27.1%	294	29.3%	280	27.9%	278	27.7%	288	28.7%	1698	28.2%
White	698	69.5%	702	70.1%	691	68.8%	697	69.5%	699	69.7%	691	68.9%	4179	69.4%
Urban	465	46.3%	459	45.8%	485	48.3%	467	46.6%	463	46.2%	474	47.3%	2812	46.7%
Suburban	382	38.0%	379	37.8%	369	36.7%	365	36.4%	376	37.5%	365	36.4%	2234	37.1%
Rural	158	15.7%	164	16.4%	151	15.0%	171	17.0%	164	16.4%	164	16.4%	969	16.1%
Separated	128	12.7%	132	13.2%	119	11.8%	122	12.2%	113	11.3%	118	11.8%	735	12.2%
Married	613	61.0%	605	60.4%	622	61.9%	634	63.2%	638	63.6%	653	65.1%	3763	62.5%
Single	251	25.0%	251	25.0%	253	25.2%	233	23.2%	239	23.8%	216	21.5%	1445	24.0%
Age 18–39	394	39.2%	389	38.8%	394	39.2%	388	38.7%	390	38.9%	392	39.1%	2348	39.0%
Age 40–59	306	30.4%	312	31.1%	307	30.5%	309	30.8%	305	30.4%	305	30.4%	1842	30.6%
Age 60+	305	30.3%	301	30.0%	304	30.2%	306	30.5%	308	30.7%	306	30.5%	1830	30.4%
Total respondents	1005	100%	1002	100%	1005	100%	1003	100%	1003	100%	1003	100%	6021	100%

**Fig. 1:**
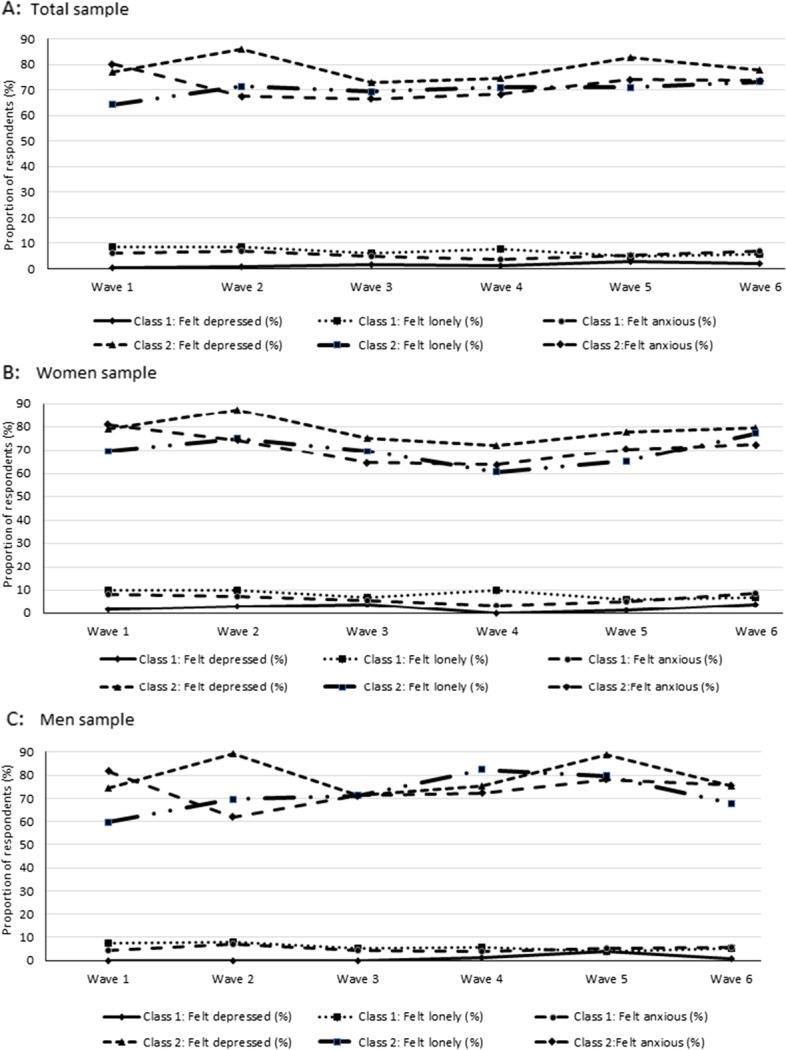
Trajectory plots for depression, anxiety, and loneliness by latent classes across waves for the total sample and by sex

Figure 1 presents the trajectories of the estimated probabilities for the mental health indicators of the latent class model across the waves. Panel A of Fig. 1 shows results for the total sample, while panels B and C show the trajectories for women and men subsample (the corresponding table is Table A.7 in the Additional file 2). The largest proportion of participants is found in Class 1 (73.9–77.1%), with around 22.9–26.1% in Class 2. Each class corresponds to an underlying subgroup of participants characterized by a particular pattern of mental health indicators during the COVID-19 pandemic. In particular, Class 2 appears to represent participants with high scores on all three mental health indicators (anxiety, feeling depressed, and feeling lonely). In this class, participants are more likely to be depressed, lonely, and anxious, with probabilities between 0.7 and 0.9, 0.6–0.7, and 0.7–0.8, respectively. As such, we will refer to Class 2 as the “high-symptom class.” In contrast, Class 1 contains participants with low scores on all three mental health indicators, who have a low probability of moderate to severe anxiety, feeling depressed, and feeling lonely (probabilities of 0.01–0.03, 0.05–0.09 and 0.04–0.07, respectively). We refer to Class 1 as the “no/low-symptoms class.” The characteristics of these two classes are consistent across the six waves. Table A. 5 displays three classes’ results: no/low-symptoms (68.9% of participants), moderate-symptoms (14%), and high-symptoms (17.1%).

Sex-specific LCA analyses were conducted to determine whether the latent classes were different for men and women. The results reported in panels B and C of Fig. 1 show that patterns for men and women are similar to those found for the entire sample, with a “no/low-symptoms” class and a “high-symptoms” class.

### Factors associated with high-symptom class membership

We used logistic regression to identify factors associated with class membership in the total sample and within each wave’s data (see Table 2). We regressed the binary variable that indicated whether individuals were in the high-symptom class based on individual and household characteristics. The adjusted odds ratios are reported in Table 2. In all six waves, individuals who worried about contracting COVID-19 were consistently at greater odds of being in the high-symptom class with aORs (95%CI) ranging between 1.72 (1.17–2.51) and 3.51 (2.20–5.60). Additionally, those aged 60 + were consistently less likely to be in this group relative to people aged less than 40 years, with aORs (95%CI) ranging between 0.26 (0.15–0.44) and 0.48 (0.29–0.77).

We used the pooled sample to test whether the risk factors for reporting a high-symptoms level of mental health varied at different time points by adding interaction terms between waves and the explanatory variables. The results are reported in the Table A.8 in the Additional file 2. From the pooled sample, we found that individuals who reported being worried about contracting COVID-19 were at greater odds of being in the high symptoms class (aOR = 2.00, 95%CI: 1.32–3.03). People aged 60+ (aOR = 0.35, 95%CI:0.22–0.56) and with a household income higher than CAD$80,000: between $80,000-$119,000 (aOR = 0.57, 95%CI:0.33–0.98) and $120,000+ (aOR = 0.37, 95%CI:0.21–0.67) were less likely than people aged less than 40 years, and people with a household income less than $40,000, to be in the high-symptoms class. Across waves the results also show some heterogeneity, suggesting that factors associated with class membership varied at different time points. In Wave 5 (relatively to Wave 6), people with children under 18 in their household (aOR = 2.20, 95%CI:1.22–3.95) and people who lived in urban area (aOR = 2.79, 95%CI:1.43–5.44) were at greater odds of being in the high symptom class than those without a child under 18 and those living in rural area respectively.

Individuals with a household income above $120,000 in Wave 4 were at greater odds of being in the high symptom class than those with a household income lower than $40,000 (aOR = 2.32, 95%CI:1.04–5.18). However, these individuals were less likely to experience a high level of mental health symptoms in the other waves than individuals in low-income households – as the other interaction coefficients were not significant. The aOR for the interaction urban*Wave 1 was 2.79 ( 95%CI:1.43–5.44) suggesting that living in urban area (relatively to rural area) were associated with a higher odds of being in the high symptoms class in Wave 1 compared to Wave 6.

**Table 2:** Factors associated with high-symptoms class membership (Adjusted Odds Ratios)

	Wave 1	Wave 2	Wave 3	Wave 4	Wave5	Wave 6
Women	1.58*** (1.16–2.15)	1.21 (0.88–1.67)	1.47** (1.07–2.02)	1.18 (0.86–1.61)	1.63*** (1.14–2.32)	1.17 (0.86–1.58)
Worry about contracting COVID-19	3.51*** (2.20–5.60)	2.42*** (1.63–3.60)	1.72*** (1.17–2.51)	2.50*** (1.66–3.75)	3.09*** (1.87–5.09)	2.00*** (1.32–3.03)
Living with others	1.07 (0.65–1.78)	0.95 (0.57–1.58)	1.55 (0.96–2.52)	0.83 (0.51–1.35)	0.74 (0.44–1.24)	1.15 (0.71–1.87)
Presence of children	1.17 (0.79–1.73)	1.16 (0.79–1.72)	0.74 (0.48–1.14)	1.27 (0.85–1.91)	1.96*** (1.26–3.05)	0.87 (0.58–1.30)
Household income less than $40,000 (ref)	-	-	-	-	-	-
Household income of $40,000-$79,999	0.73 (0.44–1.22)	0.72 (0.44–1.19)	0.69 (0.42–1.13)	0.97 (0.59–1.62)	0.55** (0.31–0.98)	0.64 (0.39–1.07)
Household income of $80,000-$119,999	0.86 (0.50–1.46)	0.40*** (0.23–0.71)	0.51** (0.30–0.87)	0.73 (0.42–1.26)	0.41*** (0.23–0.75)	0.57** (0.33–0.98)
Household income $120,000+	0.61 (0.35–1.08)	0.23*** (0.13–0.41)	0.29*** (0.16–0.53)	0.87 (0.50–1.51)	0.54** (0.29–0.99)	0.37*** (0.21–0.67)
Household income missing	0.62 (0.35–1.11)	0.39*** (0.21–0.71)	0.47** (0.26–0.84)	0.73 (0.41–1.28)	0.41*** (0.22–0.78)	0.57 (0.32–1.03)
College diploma (ref)	-	-	-	-	-	-
High school	1.11 (0.63–1.97)	0.73 (0.39–1.36)	0.86 (0.49–1.52)	1.03 (0.58–1.84)	1.81 (0.96–3.42)	0.83 (0.46–1.49)
Post-secondary	1.14 (0.69–1.88)	1.42 (0.85–2.36)	1.09 (0.64–1.86)	0.93 (0.55–1.58)	1.95** (1.07–3.58)	1.21 (0.74–1.98)
University	0.79 (0.53–1.19)	0.98 (0.64–1.49)	0.92 (0.59–1.42)	1.08 (0.72–1.62)	1.12 (0.70–1.80)	1.16 (0.79–1.72)
White (ref.)						
Non-White	1.08 (0.77–1.52)	0.87 (0.61–1.24)	1.17 (0.82–1.68)	1.18 (0.83–1.68)	1.04 (0.70–1.53)	0.92 (0.65–1.30)
Rural (ref.)						
Urban	1.42 (0.88–2.30)	1.06 (0.65–1.71)	1.21 (0.73–1.99)	0.77 (0.49–1.21)	1.68 (0.99–2.84)	0.72 (0.46–1.11)
Suburban	1.23 (0.75–2.02)	1.02 (0.62–1.68)	0.93 (0.55–1.56)	0.76 (0.48–1.22)	0.94 (0.54–1.64)	0.83 (0.53–1.30)
Single (ref.)						
Separated/ divorced/ widowed	1.30 (0.73–2.29)	1.54 (0.88–2.71)	1.91** (1.07–3.41)	0.90 (0.51–1.57)	0.64 (0.32–1.27)	1.15 (0.65–2.04)
Married	0.90 (0.57–1.41)	1.06 (0.68–1.66)	0.73 (0.46–1.16)	0.54** (0.34–0.87)	0.47*** (0.29–0.76)	0.78 (0.50–1.24)
Age 18–39 (ref.)						
Age 40–59	0.80 (0.55–1.14)	0.92 (0.63–1.33)	1.03 (0.70–1.51)	0.75 (0.51–1.10)	0.96 (0.65–1.42)	0.93 (0.65–1.33)
Age 60+	0.39*** (0.25–0.63)	0.26*** (0.15–0.44)	0.36*** (0.22–0.60)	0.48*** (0.29–0.77)	0.27*** (0.15–0.51)	0.35*** (0.22–0.56)
Constant	0.13*** (0.05–0.31)	0.39** (0.18–0.84)	0.28*** (0.12–0.61)	0.36** (0.16–0.80)	0.20*** (0.08–0.49)	0.51 (0.23–1.12)
Observations	1,002	999	1,000	1,000	1,001	997
Pseudo R-squared	0.0752	0.0972	0.0855	0.0661	0.148	0.0561

Legend: 95% confidence level in parentheses. Significance level *** *p* < 0.01, ** *p* < 0.05

### Associations of class membership with alcohol and cannabis use

The first panel of Table 3 displays associations of class membership with alcohol and cannabis use in the total sample and by sex, controlling for socio-demographic variables and worry about getting COVID-19, as well as survey wave indicator variables. Individuals in the high-symptom class had greater odds of using cannabis at least once a week and frequently engaging in heavy episodic drinking (aOR = 2.28, 95%CI:1.92–2.70; aOR = 1.71, 95%CI:1.49–1.96) relative to those in the no/low-symptoms class. Regarding changes in cannabis and alcohol consumption, results indicated that being in the high-symptom class was associated with greater odds of increasing cannabis and alcohol use during the pandemic (aOR = 3.50, 95%CI:2.80–4.37; aOR = 2.37, 95%CI:2.06–2.74). To assess whether the associations of class membership with alcohol and cannabis use are different for men and women, the second panel of Table 3 reports aORs for the interactions of class membership by sex. A significant interaction was found between class membership and sex for increase in alcohol use; the adjusted odds ratio of 0.72 (95%CI 0.54–0.95) suggests that women with high-symptoms for mental health were at lower odds of increasing the use of alcohol during the pandemic compared to men in the same class.

**Table 3:** Associations of class membership with alcohol and cannabis use (Adjusted odds ratios)

	Cannabis use	Heavy episodic drinking	Increase in cannabis use	Increase in alcohol use
Main model				
High-symptom class	2.28*** (1.92–2.70)	1.71*** (1.49–1.96)	3.50*** (2.80–4.37)	2.37*** (2.06–2.74)
Testing associations by sex				
High-symptom class	2.54*** (2.02–3.20)	1.76*** (1.45–2.14)	4.04*** (3.02–5.41)	2.83*** (2.30–3.48)
Women	0.71*** (0.58–0.87)	0.71*** (0.62–0.82)	0.73** (0.54–0.99)	1.06 (0.90–1.23)
High-symptom class*women	0.79 (0.57–1.10)	0.94 (0.72–1.23)	0.71 (0.46–1.10)	0.72** (0.54–0.95)

Odds ratios adjusted for sex, age, marital status, education, ethnicity, living area, household income, the presence of children, and other people in the household (see the Additional file 2 for the full estimation table). High-symptom class*women represents the interaction term variable between high-symptom class and women indicator variables

Legend: 95% confidence level in parentheses. Significance level *** *p* < 0.01, ** *p* < 0.05

Finally, we investigated whether the associations between class membership and cannabis and alcohol consumption varied across the survey waves or changed over time. F-tests for all coefficients of interaction terms (class membership*wave) were performed. Table 4 reports the results of these tests for the total sample, men and women. All the F-test results have *p*-values greater than 5%, except for increase in alcohol use (*p*-value < 0.05 in the total sample and among men). Overall, this suggests that the association between class membership and alcohol and cannabis use did not vary by survey wave (except for increase in alcohol drinking). This was true regardless of which wave was used as the reference (see Table A.9 in the Additional file 2). For increase in alcohol use, a significant interaction effect was found between survey waves and class membership. Compared to Wave 6, individuals in the high-symptom class were less likely to increase alcohol drinking in Wave 1 and 5. Using Wave 1 (Wave 5) as reference, confirmed that the odds of increasing alcohol drinking in people with high symptoms of mental health were greater in Wave 6 (aOR = 1.64, 95%CI:1.03–2.61 (aOR = 2.30, 95%CI: 1.39–3.79)) (see Table A. 9).

**Table 4:** Adjusted odds ratios from the model with time and latent class membership interaction

	Cannabis use past week	Heavy Episodic Drinking	Increase in cannabis use	Increase in alcohol use
Total sample				
High-symptom class	2.34*** (1.62–3.38)	2.01*** (1.46–2.76)	3.88*** (2.45–6.16)	3.39*** (2.42–4.75)
High-symptom class*wave 1	0.95 (0.55–1.65)	1.13 (0.72–1.77)	0.61 (0.30–1.22)	0.61** (0.38–0.97)
High-symptom class*wave 2	0.82 (0.47–1.42)	0.75 (0.48–1.19)	1.03 (0.51–2.04)	0.78 (0.49–1.25)
High-symptom class*wave 3	0.93 (0.53–1.61)	0.78 (0.49–1.23)	0.75 (0.37–1.53)	0.66 (0.41–1.06)
High-symptom class*wave 4	0.95 (0.55–1.65)	0.83 (0.53–1.31)	0.89 (0.44–1.81)	0.78 (0.48–1.26)
High-symptom class*wave 5	1.24 (0.71–2.15)	0.66 (0.42–1.05)	1.29 (0.61–2.74)	0.44*** (0.26–0.72)
F-test chi2 statistics	2.11	6.96	4.48	12.19
F-test *p*_value	0.833	0.223	0.483	0.0323

Odds ratios are adjusted for sex, age, marital status, education, ethnicity, living area, household income, the presence of children, and other people in the household (see the Additional file 2 for the full estimation table and the model specification)

Legend: 95% confidence level in parentheses. Significance level *** *p* < 0.01, ** *p* < 0.05

## Discussion

We applied latent class analysis to a multi-wave survey to identify classes of individuals with distinct mental health symptoms during the COVID-19 pandemic using three self-reported mental health indicators: anxiety, depression, and loneliness. We found two classes of individuals: those with high scores on all three mental health indicators and those with no/low symptoms. The two classes were consistently identified across survey waves, which suggested that the classification was robust. Individuals in the no/low symptoms class represented between 73.9 and 77.1% of participants, suggesting that a large proportion of participants reported low level of mental health symptoms. Similar class of participants was found by applying latent class growth analysis and unstructured growth mixture models on waves of an internet-based UK survey data [18, 19]. The high-symptom class was our class of interest, and it contained around 23–26% of the participants with a high probability of being anxious, feeling depressed, and feeling lonely.

The repeated cross-sectional surveys was relevant for understanding how the set of risk factors for reporting elevated mental health symptoms changes over time. For example, we found that living in urban areas increases the risk of experiencing high level of mental health symptoms in Wave 1 (May 2020) relatively to Wave 6 (November-December 2020). This may be due to the fact that the early outbreaks of COVID-19 have mostly occurred in urban areas [77], and the existence of a strong correlation between population density and COVID-19 infections [78, 79]. In addition, we identified two risk factors that was associated with Wave 5 (compared to Wave 6) of the survey – living in urban areas and having children under 18 in the household. Waves 5 and 6 (September to December 2020) were conducted during the second wave of COVID-19, with an increasing trend in the number of new infections and death by COVID-19. The fact that the provinces reopened schools for in-person learning in September 2020 (during Wave 5) may explain that living in urban areas, and having children under 18 at home were important risk factors for elevated mental health symptoms at that time. Between November and December, the number of new cases in Canada was still increasing to limit the spread of the virus some provinces reintroduced remote learning for children (e.g., Alberta, Manitoba in November 2020, Prince Edward Island in December 2020). Others like Nova Scotia started implementing their safe back-to-school plan [80]. British Columbia, Ontario, and Quebec provided schools with resources and the flexibility to offer in-person and remote learning options well before September 2020 [81]. These interventions to promote remote learning for students may explain that having children under 18 years at home was not a factor increasing the odds of being in the high-symptom class in Wave 6. However, we acknowledged that this analysis is exploratory, and evaluating the impact of government interventions is beyond the scope of this paper.

We also consistently found across the survey waves (and with the pooled sample) that individuals worried about getting COVID-19 were more likely to belong to the high-symptom class, while those aged 60 + were less likely (compared to younger adults aged less than 40) to be in this group. The latter result may be due to the negative psychological impacts of school closures on students and young parents [82, 83].

Additionally, we showed that high-symptom class membership was associated with increased odds of using cannabis and heavy episodic drinking relative to the no/low-symptoms class. Increases in cannabis as well as alcohol use were also associated with class membership. These associations did not change over time, except for increase in alcohol use.

Our first finding identifies a group of individuals who experienced high-level mental health symptoms and suggests that the well-established co-morbidity of anxiety and depression might also coexist with feelings of loneliness during the COVID-19 pandemic. This finding is consistent with previous studies demonstrating an association between loneliness, depression, anxiety and their co-morbidity [29, 30, 84]. The second main finding reveals that worrying about contracting COVID-19 (and/or fear of someone close getting COVID-19) was the only risk factor for experiencing high-level mental health symptoms that was consistent across waves and pooled data. This result reveals that as the pandemic unfolded, the fear of contracting COVID-19 was consistently associated with reporting multiple mental health symptoms, suggesting that the negative impact of the pandemic on mental health could be reduced by reducing the fear of COVID-19 within the population. Effective communication strategies employed during the pandemic from governments or public health authorities might help enhance people’s long-term psychological well-being and mitigate the fear of contracting the COVID-19 virus [85–87].

The third main finding reveals that people at high-symptoms level (compared to no/low-symptoms) were more likely to increase the use of cannabis and alcohol during the pandemic, suggesting that people with a high-symptoms level may be turning to substances to help alleviate negative symptoms. Compared to men, women with high symptoms levels were less likely to use alcohol and cannabis or increase the use of those substances during the pandemic. However, using alcohol and cannabis to deal with symptoms of anxiety and depression or with life challenges can increase the risk of developing alcohol or cannabis use disorder, or both [88]. Moreover, in the longer term, substance misuse can worsen these emotional disorder symptoms [89–91]. This implies that treatment programs are needed to better address the co-morbid disorders in response to the mental health effects of the pandemic.

The fourth main finding shows that people with high symptoms of mental health disorders were more likely to increase their alcohol drinking between November and December 2020 (Wave 6) compared to May 2020 (Wave 1) and September 2020 (Wave 5), respectively. This may be explained by the increase in daily COVID-19 cases has heightened Canadians’ fear of contracting COVID-19–45.1% and 43.3% of Canadians concerned about contracting COVID-19 in the workplace in November and December, respectively [92]. In addition, between November and December, several provinces have reintroduced stronger public health restrictions (e.g., remote learning for children, restaurants/bars closed and retail capacity limited, non-essential businesses closed, sports and recreational programming suspended, etc.) with Alberta and Ontario implemented lockdown in December [80]. This situation may have exacerbated loneliness, depression, and anxiety among adult Canadians who may use more alcohol to cope with these mental health symptoms [45–47].

Our findings confirm that mental health and associated substance use during the pandemic need attention. They suggest that initiatives (e.g., screening, virtual consultation) to improve population mental health and substance use problems during the pandemic should be adapted to account for sex and age while prioritizing men and younger adults. These initiatives should also integrate effective communication strategies to reduce people’s fear of contracting the virus and encourage behaviors that reduce the spread of COVID-19.

These findings should be considered in the context of several limitations. First, although quota sampling is the non-probability sampling method that is the closest in representativeness to probability sampling [93], its non-randomness may lead to potential selection bias [94]. However, comparing quota and probability sampling, Cumming (1990) [95] found that quota sampling with age and sex quota controls may be an acceptable alternative to probability sampling. Second, our results may not be generalizable to the general population because the surveys were performed in English only. Therefore the quota sampling was designed to be representative of English-speaking Canadians. As a result, the French-speaking population of Quebec is underrepresented in the study since the majority of the population (i.e., 85.4%) only speak French [96]. Additionally, the sampling method was not designed to provide provincial-level results, preventing us from analyzing how inter-provincial variation in alcohol and cannabis policy and regulations [97–99] and public health restrictions [80] may affect people’s use of cannabis and alcohol. Finally, cross-sectional data were collected; therefore, conclusions regarding causal relationships could not be made. Nevertheless, the study offers valuable insights into understanding mental health and substance use co-morbidities and multi-morbidities during the COVID-19 pandemic.

## Conclusions

We identified two important groups of Canadian adults during the COVID-19 pandemic: the first group with no/low levels of anxiety, depression, and loneliness, and the second with high levels of anxiety, depression, and loneliness during the COVID-19 pandemic who tended to drink more alcohol and use more cannabis compared to the first group. This finding suggests that initiatives and supports are needed to address mental health and substance use multi-morbidities, particularly during the COVID-19 pandemic.

## Supplementary Information

**Additional file 1.**

**Additional file 2.**

## Data Availability

All data generated or analyzed during this study are included in this published article [and its supplementary information files]. Data are also publicly available for download at: http://www.delvinia.com/coronavirus/.

## Abbreviations

COVID-19: Coronavirus disease 2019
LCA: Latent class analysis
GAD: generalized anxiety disorder
CES-D: Center for Epidemiologic Studies Depression
AIC: Akaike information criterion
BIC: Bayesian information criterion
aOR: Adjusted odds ratio
CI: Confidence interval
RR: Response rate
