# Supplementary material for: Self-reported mental health during the COVID-19 pandemic and its association with alcohol and cannabis use: a latent class analysis
Source: BMC Psychiatry. 2022 Apr 30;22:306. doi: 10.1186/s12888-022-03917-z (PMC9055215; doi:10.1186/s12888-022-03917-z)
Supplement: Supplementary file 2 — Additional file 2. [file 12888_2022_3917_MOESM2_ESM.docx]

**APPENDIX**

**Models specification**

The general form of the estimating equation was

$$y_{iw}=\beta_{0}+\beta_{1}{LC}_{i}+\sum_{w=1}^{5} \phi_{w}D_{w}+{\gamma'Char}_{iw}+{\varphi'Hc}_{iw}+u_{it} (1)$$

Where $y_{iw}$ is the one if the participant $i$ was a heavy episodic drinker (or use cannabis at least once a week) in wave $w=$ 1 to 6. ${Char}_{iw}$ is a vector of individual $i$ characteristics, ${Hc}_{iw}$ is a vector of individual $i$ household characteristics in wave $w$, and $D_{w}$ is an indicator variable for each wave $w$ (with wave 6 as reference). Our parameter of interest is $\beta_{1}$, the coefficient of the latent class variable (${LC}_{i}$). We used logistic regression to estimate $\beta_{1}$; it measures the relatively higher or lower odds of engaging in heavy episodic drinking or consuming cannabis at least once in the previous week during the pandemic for those in the latent class of interest as compared to those in other latent classes. We also investigated whether the effect of class membership varied through time by adding interactions between ${LC}_{i}$ and survey wave binary variables ($D_{w}$). The estimating equation specification changed to

$$y_{iw}=\beta_{0}+\beta_{1}{LC}_{i}+\sum_{w=1}^{5} \beta_{w+1}{LC}_{i}*D_{w}+\sum_{w=1}^{5} \phi_{w}D_{w}+{\gamma'Char}_{iw}+{\varphi'Hc}_{iw}+u_{it} (2)$$

where the parameters of interest were $\beta_{w}$ with $w=$ 1 to 6. We then calculated the F-test for the joint significance of interaction terms to detect time/wave effects.

To test if the odds ratios across waves are all statistically equal, we tested the null hypothesis that all the coefficients of the interaction terms are zero using an F-test ($H_{0}: \beta_{2}=\beta_{3}=\beta_{4}=\beta_{5}=\beta_{6}=0$) using separately the total sample and men and women subsamples.

Table A.1: Survey interviews information and response rate calculations

|  | **WAVE 1** *[May 8-12]* | **WAVE 2** *[May 29-Jun 1]* | **WAVE 3** *[June 19-23]* | **WAVE 4** *[July 10-14]* | **WAVE 5** *[Sept 18-22]* | **WAVE 6** *[Nov 27-Dec 1]* | ***Pooled Data*: WAVES 1-6** |
| --- | --- | --- | --- | --- | --- | --- | --- |
| ***INTERVIEWS*** | *Total* | *Total* | *Total* | *Total* | *Total* | *Total* | *Total* |
| Complete | 1005 | 1002 | 1005 | 1003 | 1003 | 1003 | 6021 |
| Incomplete | 109 | 129 | 114 | 112 | 138 | 124 | 726 |
| Screened | 65 | 89 | 76 | 100 | 112 | 87 | 529 |
| QuotaFull | 203 | 213 | 131 | 150 | 145 | 135 | 977 |
| Error | 0 | 0 | 0 | 0 | 0 | 0 | 0 |
| Total interviews started (*sum of previous cells*) | 1382 | 1433 | 1326 | 1365 | 1398 | 1349 | 8253 |
| Invites sent | 6577 | 6130 | 6336 | 7575 | 5962 | 6407 | 38987 |
| ***RESPONSE RATE*** | ***%*** | ***%*** | ***%*** | ***%*** | ***%*** | ***%*** | ***%*** |
| RR based on #completed interviews/estimated # eligible respondents [i.e., completed / (sent-quotafull-screened)] | 15.93 | 17.19 | 16.4 | 13.69 | 17.58 | 16.22 | 16.06 |

Table A.2: Multivariate logistic regression models of alcohol and cannabis use during the pandemic (Adjusted odds ratios)

|  | **All** | | | | **Women** | | | | **Men** | | | |
| --- | --- | --- | --- | --- | --- | --- | --- | --- | --- | --- | --- | --- |
| VARIABLES | Cannabis use past week | Heavy Episodic Drinking | Increase in cannabis use | Increase in alcohol use | Cannabis use past week | Heavy Episodic Drinking | Increase in cannabis use | Increase in alcohol use | Cannabis use past week | Heavy Episodic Drinking | Increase in cannabis use | Increase in alcohol use |
|  |  |  |  |  |  |  |  |  |  |  |  |  |
| High-symptoms class | 2.28*** | 1.71*** | 3.50*** | 2.37*** | 1.98*** | 1.68*** | 2.88*** | 2.11*** | 2.56*** | 1.73*** | 3.91*** | 2.63*** |
|  | (1.92 - 2.70) | (1.49 - 1.96) | (2.80 - 4.37) | (2.06 - 2.74) | (1.54 - 2.54) | (1.39 - 2.05) | (2.05 - 4.04) | (1.73 - 2.57) | (2.02 - 3.25) | (1.41 - 2.11) | (2.88 - 5.29) | (2.12 - 3.25) |
| Worrying about COVID-19 | 0.89 | 1.09 | 1.03 | 1.26*** | 0.93 | 1.04 | 0.81 | 1.11 | 0.88 | 1.12 | 1.28 | 1.38*** |
|  | (0.74 - 1.07) | (0.95 - 1.26) | (0.79 - 1.34) | (1.08 - 1.47) | (0.70 - 1.25) | (0.84 - 1.29) | (0.55 - 1.19) | (0.89 - 1.38) | (0.69 - 1.12) | (0.93 - 1.36) | (0.89 - 1.84) | (1.11 - 1.71) |
| Living with others | 1.20 | 1.00 | 1.17 | 1.00 | 0.91 | 1.01 | 0.77 | 0.92 | 1.45** | 0.99 | 1.51 | 1.18 |
|  | (0.94 - 1.54) | (0.82 - 1.22) | (0.82 - 1.65) | (0.80 - 1.24) | (0.63 - 1.31) | (0.76 - 1.35) | (0.46 - 1.29) | (0.68 - 1.24) | (1.02 - 2.06) | (0.75 - 1.32) | (0.92 - 2.46) | (0.85 - 1.63) |
| Presence of children | 0.98 | 0.97 | 1.07 | 1.06 | 0.97 | 0.97 | 1.11 | 0.92 | 0.94 | 0.99 | 0.91 | 1.20 |
|  | (0.81 - 1.19) | (0.84 - 1.14) | (0.83 - 1.37) | (0.90 - 1.25) | (0.72 - 1.31) | (0.77 - 1.22) | (0.76 - 1.63) | (0.73 - 1.17) | (0.71 - 1.23) | (0.80 - 1.22) | (0.65 - 1.29) | (0.95 - 1.50) |
| Income less than $40,000 | REF | REF | REF | REF | REF | REF | REF | REF | REF | REF | REF | REF |
| Income in $40,000-$79,999 | 1.10 | 1.54*** | 1.15 | 1.42*** | 1.16 | 1.53*** | 1.07 | 1.51** | 0.99 | 1.58*** | 1.15 | 1.37* |
|  | (0.85 - 1.43) | (1.24 - 1.92) | (0.80 - 1.64) | (1.10 - 1.81) | (0.79 - 1.70) | (1.12 - 2.09) | (0.64 - 1.81) | (1.07 - 2.15) | (0.68 - 1.43) | (1.16 - 2.17) | (0.69 - 1.92) | (0.95 - 1.96) |
| Income in $80,000-$119,999 | 1.14 | 1.52*** | 1.29 | 1.79*** | 1.28 | 1.51** | 1.53 | 2.05*** | 1.02 | 1.60*** | 1.16 | 1.68*** |
|  | (0.86 - 1.50) | (1.21 - 1.91) | (0.89 - 1.87) | (1.39 - 2.31) | (0.84 - 1.94) | (1.08 - 2.11) | (0.88 - 2.66) | (1.43 - 2.94) | (0.70 - 1.49) | (1.16 - 2.20) | (0.69 - 1.94) | (1.17 - 2.41) |
| Income $120,000+ | 0.76* | 1.57*** | 0.78 | 2.09*** | 0.70 | 1.46** | 0.85 | 2.12*** | 0.75 | 1.71*** | 0.70 | 2.15*** |
|  | (0.57 - 1.02) | (1.24 - 1.99) | (0.52 - 1.17) | (1.62 - 2.71) | (0.44 - 1.12) | (1.03 - 2.06) | (0.45 - 1.60) | (1.46 - 3.06) | (0.50 - 1.11) | (1.23 - 2.37) | (0.40 - 1.23) | (1.49 - 3.11) |
| Income missing | 0.55*** | 0.93 | 0.37*** | 1.24 | 0.65* | 0.85 | 0.46** | 1.39* | 0.44*** | 1.11 | 0.30*** | 1.15 |
|  | (0.39 - 0.77) | (0.73 - 1.20) | (0.22 - 0.62) | (0.94 - 1.63) | (0.41 - 1.03) | (0.60 - 1.21) | (0.23 - 0.93) | (0.96 - 2.01) | (0.26 - 0.74) | (0.76 - 1.61) | (0.14 - 0.67) | (0.75 - 1.77) |
| College diploma | REF | REF | REF | REF | REF | REF | REF | REF | REF | REF | REF | REF |
| High school | 1.01 | 1.15 | 1.18 | 0.67*** | 0.88 | 0.97 | 1.02 | 0.51*** | 1.14 | 1.30* | 1.28 | 0.89 |
|  | (0.77 - 1.34) | (0.92 - 1.43) | (0.78 - 1.77) | (0.52 - 0.87) | (0.59 - 1.32) | (0.71 - 1.34) | (0.56 - 1.87) | (0.35 - 0.74) | (0.77 - 1.71) | (0.96 - 1.77) | (0.71 - 2.29) | (0.62 - 1.30) |
| Post-secondary | 1.02 | 1.07 | 1.67*** | 0.86 | 0.81 | 0.89 | 1.43 | 0.73** | 1.27 | 1.25 | 1.94*** | 1.06 |
|  | (0.79 - 1.31) | (0.88 - 1.30) | (1.19 - 2.35) | (0.70 - 1.07) | (0.56 - 1.19) | (0.66 - 1.19) | (0.85 - 2.40) | (0.53 - 0.99) | (0.89 - 1.81) | (0.95 - 1.64) | (1.20 - 3.12) | (0.78 - 1.45) |
| University | 0.62*** | 0.83** | 0.85 | 1.04 | 0.51*** | 0.91 | 0.76 | 1.06 | 0.74** | 0.76** | 0.93 | 1.04 |
|  | (0.51 - 0.77) | (0.71 - 0.97) | (0.63 - 1.14) | (0.88 - 1.23) | (0.38 - 0.69) | (0.72 - 1.14) | (0.49 - 1.17) | (0.84 - 1.33) | (0.55 - 0.98) | (0.61 - 0.95) | (0.62 - 1.40) | (0.82 - 1.33) |
| White | REF | REF | REF | REF | REF | REF | REF | REF | REF | REF | REF | REF |
| Non-white | 0.79*** | 0.89* | 0.92 | 0.67*** | 0.89 | 0.87 | 1.03 | 0.63*** | 0.69*** | 0.90 | 0.80 | 0.67*** |
|  | (0.66 - 0.94) | (0.77 - 1.02) | (0.72 - 1.18) | (0.57 - 0.78) | (0.67 - 1.18) | (0.70 - 1.07) | (0.71 - 1.51) | (0.50 - 0.79) | (0.54 - 0.88) | (0.74 - 1.09) | (0.57 - 1.11) | (0.54 - 0.84) |
| Suburban | REF | REF | REF | REF | REF | REF | REF | REF | REF | REF | REF | REF |
| Urban | 1.09 | 0.94 | 0.97 | 1.04 | 0.95 | 0.84* | 0.93 | 0.91 | 1.17 | 1.03 | 0.97 | 1.20* |
|  | (0.91 - 1.30) | (0.82 - 1.07) | (0.77 - 1.22) | (0.90 - 1.20) | (0.72 - 1.26) | (0.69 - 1.03) | (0.64 - 1.35) | (0.74 - 1.12) | (0.93 - 1.47) | (0.86 - 1.23) | (0.72 - 1.32) | (0.98 - 1.46) |
| Rural | 0.99 | 0.88 | 0.81 | 0.84* | 1.33* | 0.85 | 1.16 | 0.75** | 0.63** | 0.90 | 0.45*** | 0.92 |
|  | (0.78 - 1.27) | (0.73 - 1.06) | (0.57 - 1.16) | (0.68 - 1.02) | (0.96 - 1.86) | (0.66 - 1.11) | (0.72 - 1.87) | (0.57 - 0.98) | (0.42 - 0.93) | (0.69 - 1.18) | (0.25 - 0.81) | (0.67 - 1.25) |
| Women | 0.66*** | 0.71*** | 0.63*** | 0.96 | - | - | - | - | - | - | - | - |
|  | (0.56 - 0.78) | (0.63 - 0.80) | (0.50 - 0.78) | (0.84 - 1.09) |  |  |  |  |  |  |  |  |
| Age 18-39 | REF | REF | REF | REF | REF | REF | REF | REF | REF | REF | REF | REF |
| Age 40-59 | 0.54*** | 0.88* | 0.48*** | 0.96 | 0.47*** | 0.88 | 0.40*** | 0.96 | 0.59*** | 0.85 | 0.50*** | 0.93 |
|  | (0.44 - 0.65) | (0.76 - 1.01) | (0.37 - 0.62) | (0.82 - 1.13) | (0.35 - 0.63) | (0.71 - 1.10) | (0.26 - 0.60) | (0.77 - 1.20) | (0.45 - 0.76) | (0.70 - 1.05) | (0.35 - 0.70) | (0.75 - 1.17) |
| Age 60+ | 0.23*** | 0.57*** | 0.13*** | 0.69*** | 0.19*** | 0.59*** | 0.09*** | 0.70** | 0.26*** | 0.55*** | 0.14*** | 0.64*** |
|  | (0.18 - 0.31) | (0.47 - 0.68) | (0.09 - 0.21) | (0.57 - 0.83) | (0.12 - 0.29) | (0.44 - 0.78) | (0.04 - 0.21) | (0.54 - 0.93) | (0.18 - 0.37) | (0.43 - 0.70) | (0.08 - 0.24) | (0.49 - 0.85) |
| Single | REF | REF | REF | REF | REF | REF | REF | REF | REF | REF | REF | REF |
| Separated | 1.36** | 1.22* | 1.19 | 1.18 | 1.44 | 1.14 | 1.32 | 1.36* | 1.18 | 1.29 | 1.06 | 0.95 |
|  | (1.00 - 1.85) | (0.97 - 1.54) | (0.76 - 1.86) | (0.91 - 1.53) | (0.93 - 2.24) | (0.82 - 1.58) | (0.71 - 2.48) | (0.96 - 1.92) | (0.74 - 1.88) | (0.91 - 1.82) | (0.52 - 2.17) | (0.63 - 1.43) |
| Married | 1.03 | 1.17* | 1.13 | 1.30*** | 1.07 | 1.11 | 1.08 | 1.45*** | 0.96 | 1.22 | 1.24 | 1.13 |
|  | (0.82 - 1.29) | (0.98 - 1.40) | (0.84 - 1.53) | (1.07 - 1.59) | (0.76 - 1.49) | (0.85 - 1.44) | (0.70 - 1.66) | (1.10 - 1.92) | (0.69 - 1.32) | (0.94 - 1.58) | (0.80 - 1.92) | (0.85 - 1.52) |
| Wave 6 | REF | REF | REF | REF | REF | REF | REF | REF | REF | REF | REF | REF |
| Wave 1 | 0.65*** | 0.91 | 0.62*** | 1.36*** | 0.72 | 0.85 | 0.63* | 1.36** | 0.64** | 0.99 | 0.66* | 1.41** |
|  | (0.49 - 0.84) | (0.74 - 1.11) | (0.44 - 0.88) | (1.10 - 1.68) | (0.47 - 1.10) | (0.63 - 1.16) | (0.37 - 1.08) | (1.01 - 1.84) | (0.45 - 0.92) | (0.75 - 1.31) | (0.41 - 1.05) | (1.03 - 1.92) |
| Wave 2 | 0.76** | 0.95 | 0.72* | 1.30** | 0.98 | 0.72** | 0.74 | 1.05 | 0.63** | 1.19 | 0.70 | 1.58*** |
|  | (0.59 - 0.99) | (0.78 - 1.17) | (0.51 - 1.02) | (1.05 - 1.61) | (0.66 - 1.46) | (0.53 - 0.99) | (0.44 - 1.25) | (0.77 - 1.44) | (0.44 - 0.92) | (0.90 - 1.58) | (0.44 - 1.12) | (1.16 - 2.15) |
| Wave 3 | 0.75** | 1.09 | 0.62*** | 1.13 | 1.00 | 1.18 | 0.63* | 1.08 | 0.62*** | 1.03 | 0.62* | 1.20 |
|  | (0.57 - 0.97) | (0.89 - 1.33) | (0.43 - 0.88) | (0.91 - 1.41) | (0.67 - 1.49) | (0.88 - 1.58) | (0.37 - 1.09) | (0.79 - 1.47) | (0.43 - 0.89) | (0.78 - 1.37) | (0.38 - 1.01) | (0.88 - 1.65) |
| Wave 4 | 0.79* | 1.11 | 0.66** | 1.08 | 0.80 | 1.19 | 0.49** | 0.97 | 0.82 | 1.09 | 0.83 | 1.22 |
|  | (0.61 - 1.03) | (0.91 - 1.36) | (0.46 - 0.93) | (0.86 - 1.35) | (0.53 - 1.22) | (0.89 - 1.61) | (0.27 - 0.89) | (0.70 - 1.33) | (0.58 - 1.16) | (0.82 - 1.44) | (0.53 - 1.31) | (0.89 - 1.67) |
| Wave 5 | 0.70*** | 1.01 | 0.52*** | 0.79** | 0.85 | 0.99 | 0.58* | 0.77 | 0.60*** | 1.06 | 0.50*** | 0.82 |
|  | (0.54 - 0.92) | (0.82 - 1.24) | (0.36 - 0.76) | (0.62 - 0.99) | (0.57 - 1.27) | (0.73 - 1.34) | (0.34 - 1.00) | (0.56 - 1.06) | (0.42 - 0.87) | (0.79 - 1.40) | (0.30 - 0.83) | (0.59 - 1.15) |
| Constant | 0.39*** | 0.31*** | 0.12*** | 0.12*** | 0.31*** | 0.26*** | 0.15*** | 0.15*** | 0.36*** | 0.25*** | 0.08*** | 0.08*** |
|  | (0.26 - 0.60) | (0.22 - 0.43) | (0.07 - 0.22) | (0.08 - 0.17) | (0.17 - 0.58) | (0.16 - 0.42) | (0.07 - 0.34) | (0.09 - 0.24) | (0.20 - 0.64) | (0.16 - 0.40) | (0.04 - 0.19) | (0.05 - 0.14) |
| Observations | 5,986 | 5,982 | 5,999 | 5,999 | 2,973 | 2,971 | 2,976 | 2,976 | 2,970 | 2,968 | 2,980 | 2,980 |
| Pseudo R-squared | 0.0867 | 0.0324 | 0.141 | 0.0534 | 0.0908 | 0.0324 | 0.135 | 0.0542 | 0.0912 | 0.0305 | 0.154 | 0.0621 |
| Legend: 95% confidence level in parentheses. Significance level *** p<0.01, ** p<0.05, * p<0.1 | | | | | | | | | | | | |

Table A.3: Multivariate logistic regression models of alcohol and cannabis use with latent class-wave interactions (Adjusted odds ratios)

|  | **All** | | | | **Women** | | | | **Men** | | | |
| --- | --- | --- | --- | --- | --- | --- | --- | --- | --- | --- | --- | --- |
| VARIABLES | Cannabis use past week | Heavy Episodic Drinking | Increase in cannabis use | Increase in alcohol use | Cannabis use past week | Heavy Episodic Drinking | Increase in cannabis use | Increase in alcohol use | Cannabis use past week | Heavy Episodic Drinking | Increase in cannabis use | Increase in alcohol use |
| High-symptoms class | 2.34*** | 2.01*** | 3.88*** | 3.39*** | 1.99** | 1.68** | 3.13*** | 2.31*** | 2.51*** | 2.25*** | 4.15*** | 4.76*** |
|  | (1.62 - 3.38) | (1.46 - 2.76) | (2.45 - 6.16) | (2.42 - 4.75) | (1.12 - 3.54) | (1.07 - 2.64) | (1.54 - 6.38) | (1.45 - 3.69) | (1.52 - 4.17) | (1.42 - 3.57) | (2.22 - 7.78) | (2.88 - 7.85) |
| High-symptoms class*wave 1 | 0.95 | 1.13 | 0.61 | 0.61** | 0.99 | 1.36 | 0.42 | 0.78 | 1.02 | 1.07 | 0.85 | 0.52* |
|  | (0.55 - 1.65) | (0.72 - 1.77) | (0.30 - 1.22) | (0.38 - 0.97) | (0.43 - 2.32) | (0.71 - 2.57) | (0.15 - 1.20) | (0.41 - 1.47) | (0.48 - 2.17) | (0.56 - 2.05) | (0.33 - 2.17) | (0.26 - 1.05) |
| High-symptoms class*wave 2 | 0.82 | 0.75 | 1.03 | 0.78 | 0.91 | 0.96 | 1.17 | 0.76 | 0.68 | 0.63 | 0.92 | 0.79 |
|  | (0.47 - 1.42) | (0.48 - 1.19) | (0.51 - 2.04) | (0.49 - 1.25) | (0.40 - 2.06) | (0.49 - 1.89) | (0.41 - 3.39) | (0.39 - 1.48) | (0.31 - 1.51) | (0.32 - 1.22) | (0.36 - 2.39) | (0.40 - 1.58) |
| High-symptoms class*wave 3 | 0.93 | 0.78 | 0.75 | 0.66* | 0.73 | 0.99 | 0.69 | 1.16 | 1.22 | 0.59 | 0.90 | 0.36*** |
|  | (0.53 - 1.61) | (0.49 - 1.23) | (0.37 - 1.53) | (0.41 - 1.06) | (0.32 - 1.67) | (0.52 - 1.86) | (0.23 - 2.05) | (0.60 - 2.24) | (0.56 - 2.65) | (0.30 - 1.17) | (0.34 - 2.35) | (0.17 - 0.74) |
| High-symptoms class*wave 4 | 0.95 | 0.83 | 0.89 | 0.78 | 1.02 | 0.98 | 1.09 | 1.24 | 1.05 | 0.75 | 0.91 | 0.52* |
|  | (0.55 - 1.65) | (0.53 - 1.31) | (0.44 - 1.81) | (0.48 - 1.26) | (0.43 - 2.44) | (0.51 - 1.87) | (0.33 - 3.64) | (0.62 - 2.46) | (0.50 - 2.19) | (0.38 - 1.45) | (0.37 - 2.28) | (0.26 - 1.04) |
| High-symptoms class*wave 5 | 1.24 | 0.66* | 1.29 | 0.44*** | 1.45 | 0.78 | 1.82 | 0.66 | 1.27 | 0.64 | 1.08 | 0.30*** |
|  | (0.71 - 2.15) | (0.42 - 1.05) | (0.61 - 2.74) | (0.26 - 0.72) | (0.63 - 3.32) | (0.41 - 1.50) | (0.56 - 5.92) | (0.33 - 1.33) | (0.59 - 2.74) | (0.33 - 1.24) | (0.40 - 2.97) | (0.14 - 0.64) |
| Worrying about COVID-19 | 0.89 | 1.09 | 1.04 | 1.26*** | 0.93 | 1.04 | 0.79 | 1.12 | 0.88 | 1.12 | 1.28 | 1.39*** |
|  | (0.74 - 1.07) | (0.95 - 1.26) | (0.80 - 1.35) | (1.08 - 1.47) | (0.69 - 1.24) | (0.84 - 1.29) | (0.54 - 1.17) | (0.89 - 1.40) | (0.69 - 1.12) | (0.93 - 1.36) | (0.89 - 1.85) | (1.12 - 1.72) |
| Living with others | 1.20 | 1.00 | 1.17 | 1.00 | 0.92 | 1.01 | 0.79 | 0.92 | 1.44** | 0.99 | 1.50 | 1.19 |
|  | (0.94 - 1.54) | (0.82 - 1.22) | (0.83 - 1.65) | (0.80 - 1.24) | (0.64 - 1.33) | (0.76 - 1.34) | (0.47 - 1.33) | (0.68 - 1.24) | (1.01 - 2.04) | (0.75 - 1.31) | (0.92 - 2.46) | (0.86 - 1.66) |
| Presence of children | 0.98 | 0.98 | 1.06 | 1.07 | 0.97 | 0.97 | 1.10 | 0.93 | 0.94 | 0.99 | 0.91 | 1.20 |
|  | (0.80 - 1.19) | (0.84 - 1.14) | (0.83 - 1.37) | (0.91 - 1.26) | (0.71 - 1.30) | (0.77 - 1.22) | (0.75 - 1.61) | (0.73 - 1.18) | (0.72 - 1.24) | (0.80 - 1.22) | (0.64 - 1.28) | (0.95 - 1.51) |
| Income less than $40,000 | REF | REF | REF | REF | REF | REF | REF | REF | REF | REF | REF | REF |
| Income in $40,000-$79,999 | 1.10 | 1.54*** | 1.15 | 1.41*** | 1.16 | 1.53*** | 1.06 | 1.51** | 1.00 | 1.57*** | 1.16 | 1.34 |
|  | (0.85 - 1.44) | (1.24 - 1.92) | (0.80 - 1.65) | (1.10 - 1.80) | (0.79 - 1.70) | (1.12 - 2.10) | (0.63 - 1.81) | (1.06 - 2.14) | (0.69 - 1.44) | (1.14 - 2.15) | (0.69 - 1.93) | (0.93 - 1.92) |
| Income in $80,000-$119,999 | 1.14 | 1.51*** | 1.31 | 1.79*** | 1.27 | 1.50** | 1.56 | 2.05*** | 1.03 | 1.58*** | 1.16 | 1.65*** |
|  | (0.86 - 1.50) | (1.20 - 1.89) | (0.90 - 1.89) | (1.39 - 2.30) | (0.83 - 1.93) | (1.07 - 2.09) | (0.90 - 2.73) | (1.43 - 2.94) | (0.71 - 1.50) | (1.15 - 2.18) | (0.69 - 1.95) | (1.15 - 2.38) |
| Income $120,000+ | 0.76* | 1.56*** | 0.78 | 2.10*** | 0.69 | 1.46** | 0.85 | 2.13*** | 0.74 | 1.68*** | 0.71 | 2.14*** |
|  | (0.56 - 1.02) | (1.23 - 1.98) | (0.52 - 1.18) | (1.63 - 2.72) | (0.43 - 1.10) | (1.03 - 2.06) | (0.45 - 1.61) | (1.47 - 3.07) | (0.50 - 1.11) | (1.21 - 2.33) | (0.40 - 1.24) | (1.48 - 3.10) |
| Income missing | 0.55*** | 0.93 | 0.37*** | 1.24 | 0.64* | 0.85 | 0.46** | 1.39* | 0.44*** | 1.09 | 0.31*** | 1.14 |
|  | (0.39 - 0.77) | (0.72 - 1.20) | (0.22 - 0.63) | (0.94 - 1.62) | (0.41 - 1.02) | (0.60 - 1.21) | (0.23 - 0.92) | (0.96 - 2.00) | (0.26 - 0.74) | (0.75 - 1.59) | (0.14 - 0.68) | (0.74 - 1.75) |
| College diploma | REF | REF | REF | REF | REF | REF | REF | REF | REF | REF | REF | REF |
| High school | 1.01 | 1.15 | 1.18 | 0.68*** | 0.87 | 0.98 | 0.99 | 0.51*** | 1.14 | 1.29 | 1.27 | 0.88 |
|  | (0.76 - 1.33) | (0.92 - 1.43) | (0.78 - 1.77) | (0.52 - 0.88) | (0.58 - 1.31) | (0.71 - 1.35) | (0.54 - 1.82) | (0.35 - 0.75) | (0.76 - 1.70) | (0.95 - 1.76) | (0.71 - 2.28) | (0.61 - 1.28) |
| Post-secondary | 1.01 | 1.07 | 1.67*** | 0.87 | 0.81 | 0.89 | 1.43 | 0.74* | 1.27 | 1.24 | 1.93*** | 1.05 |
|  | (0.79 - 1.30) | (0.88 - 1.30) | (1.19 - 2.34) | (0.70 - 1.08) | (0.55 - 1.18) | (0.66 - 1.20) | (0.85 - 2.39) | (0.54 - 1.00) | (0.89 - 1.81) | (0.94 - 1.63) | (1.20 - 3.11) | (0.77 - 1.44) |
| University | 0.62*** | 0.83** | 0.84 | 1.04 | 0.51*** | 0.91 | 0.75 | 1.06 | 0.74** | 0.76** | 0.93 | 1.03 |
|  | (0.51 - 0.77) | (0.71 - 0.97) | (0.63 - 1.13) | (0.88 - 1.23) | (0.38 - 0.69) | (0.72 - 1.15) | (0.48 - 1.15) | (0.85 - 1.34) | (0.55 - 0.98) | (0.61 - 0.95) | (0.62 - 1.40) | (0.81 - 1.32) |
| White | REF | REF | REF | REF | REF | REF | REF | REF | REF | REF | REF | REF |
| Non-white | 0.79*** | 0.89* | 0.93 | 0.67*** | 0.89 | 0.86 | 1.04 | 0.62*** | 0.68*** | 0.90 | 0.80 | 0.68*** |
|  | (0.66 - 0.94) | (0.77 - 1.02) | (0.72 - 1.19) | (0.57 - 0.78) | (0.67 - 1.18) | (0.70 - 1.06) | (0.71 - 1.53) | (0.50 - 0.78) | (0.53 - 0.87) | (0.74 - 1.09) | (0.57 - 1.12) | (0.55 - 0.85) |
| Suburban | REF | REF | REF | REF | REF | REF | REF | REF | REF | REF | REF | REF |
| Urban | 1.09 | 0.94 | 0.97 | 1.05 | 0.95 | 0.85* | 0.92 | 0.92 | 1.16 | 1.03 | 0.97 | 1.21* |
|  | (0.91 - 1.29) | (0.83 - 1.07) | (0.77 - 1.22) | (0.91 - 1.21) | (0.72 - 1.25) | (0.69 - 1.03) | (0.63 - 1.34) | (0.75 - 1.13) | (0.92 - 1.47) | (0.86 - 1.24) | (0.72 - 1.32) | (0.99 - 1.48) |
| Rural | 1.00 | 0.88 | 0.81 | 0.83* | 1.34* | 0.85 | 1.18 | 0.74** | 0.63** | 0.90 | 0.45*** | 0.91 |
|  | (0.78 - 1.28) | (0.73 - 1.06) | (0.57 - 1.15) | (0.68 - 1.01) | (0.96 - 1.87) | (0.66 - 1.10) | (0.73 - 1.90) | (0.57 - 0.97) | (0.42 - 0.94) | (0.69 - 1.18) | (0.25 - 0.81) | (0.67 - 1.24) |
| Women | 0.66*** | 0.71*** | 0.63*** | 0.96 | - | - | - | - | - | - | - | - |
|  | (0.56 - 0.78) | (0.62 - 0.80) | (0.50 - 0.79) | (0.84 - 1.09) |  |  |  |  |  |  |  |  |
| Age 18-39 |  |  |  |  |  |  |  |  |  |  |  |  |
| Age 40-59 | 0.54*** | 0.88* | 0.48*** | 0.96 | 0.47*** | 0.89 | 0.39*** | 0.96 | 0.58*** | 0.85 | 0.50*** | 0.93 |
|  | (0.44 - 0.65) | (0.76 - 1.01) | (0.37 - 0.62) | (0.82 - 1.12) | (0.35 - 0.63) | (0.72 - 1.10) | (0.26 - 0.59) | (0.77 - 1.20) | (0.45 - 0.76) | (0.70 - 1.04) | (0.35 - 0.71) | (0.75 - 1.17) |
| Age 60+ | 0.23*** | 0.57*** | 0.13*** | 0.69*** | 0.19*** | 0.59*** | 0.10*** | 0.70** | 0.26*** | 0.54*** | 0.14*** | 0.65*** |
|  | (0.18 - 0.31) | (0.47 - 0.68) | (0.09 - 0.21) | (0.57 - 0.83) | (0.12 - 0.29) | (0.44 - 0.77) | (0.04 - 0.21) | (0.53 - 0.92) | (0.18 - 0.37) | (0.43 - 0.69) | (0.08 - 0.24) | (0.49 - 0.85) |
| Single | REF | REF | REF | REF | REF | REF | REF | REF | REF | REF | REF | REF |
| Separated | 1.37** | 1.21 | 1.20 | 1.17 | 1.47* | 1.14 | 1.33 | 1.34* | 1.19 | 1.28 | 1.07 | 0.94 |
|  | (1.01 - 1.87) | (0.96 - 1.53) | (0.77 - 1.87) | (0.90 - 1.51) | (0.95 - 2.28) | (0.82 - 1.57) | (0.71 - 2.49) | (0.95 - 1.90) | (0.74 - 1.89) | (0.90 - 1.81) | (0.52 - 2.18) | (0.62 - 1.42) |
| Married | 1.03 | 1.16 | 1.14 | 1.29** | 1.07 | 1.10 | 1.06 | 1.44** | 0.97 | 1.21 | 1.24 | 1.12 |
|  | (0.82 - 1.30) | (0.97 - 1.39) | (0.84 - 1.54) | (1.06 - 1.57) | (0.77 - 1.49) | (0.85 - 1.44) | (0.69 - 1.65) | (1.09 - 1.91) | (0.70 - 1.34) | (0.94 - 1.57) | (0.80 - 1.93) | (0.83 - 1.49) |
| Wave 6 | REF | REF | REF | REF | REF | REF | REF | REF | REF | REF | REF | REF |
| Wave 1 | 0.66** | 0.88 | 0.79 | 1.61*** | 0.72 | 0.76 | 0.95 | 1.48** | 0.64** | 0.98 | 0.71 | 1.76*** |
|  | (0.47 - 0.92) | (0.68 - 1.12) | (0.49 - 1.26) | (1.23 - 2.10) | (0.42 - 1.26) | (0.52 - 1.12) | (0.47 - 1.94) | (1.02 - 2.14) | (0.41 - 0.98) | (0.71 - 1.37) | (0.38 - 1.33) | (1.19 - 2.59) |
| Wave 2 | 0.82 | 1.03 | 0.72 | 1.42** | 1.02 | 0.73 | 0.69 | 1.15 | 0.72 | 1.34* | 0.73 | 1.77*** |
|  | (0.59 - 1.14) | (0.81 - 1.32) | (0.44 - 1.16) | (1.09 - 1.86) | (0.61 - 1.69) | (0.50 - 1.08) | (0.32 - 1.45) | (0.79 - 1.68) | (0.46 - 1.10) | (0.97 - 1.85) | (0.38 - 1.37) | (1.20 - 2.61) |
| Wave 3 | 0.77 | 1.17 | 0.71 | 1.31* | 1.12 | 1.18 | 0.75 | 1.03 | 0.58** | 1.17 | 0.65 | 1.63** |
|  | (0.55 - 1.06) | (0.92 - 1.48) | (0.44 - 1.14) | (1.00 - 1.71) | (0.67 - 1.85) | (0.83 - 1.69) | (0.36 - 1.57) | (0.70 - 1.52) | (0.37 - 0.91) | (0.84 - 1.61) | (0.34 - 1.26) | (1.11 - 2.40) |
| Wave 4 | 0.81 | 1.17 | 0.69 | 1.18 | 0.80 | 1.20 | 0.47* | 0.91 | 0.81 | 1.17 | 0.86 | 1.52** |
|  | (0.59 - 1.11) | (0.92 - 1.49) | (0.43 - 1.12) | (0.90 - 1.56) | (0.47 - 1.36) | (0.84 - 1.72) | (0.20 - 1.08) | (0.61 - 1.35) | (0.54 - 1.22) | (0.85 - 1.62) | (0.47 - 1.58) | (1.03 - 2.26) |
| Wave 5 | 0.64** | 1.14 | 0.45*** | 1.03 | 0.72 | 1.07 | 0.40** | 0.88 | 0.56*** | 1.18 | 0.48** | 1.19 |
|  | (0.46 - 0.91) | (0.89 - 1.45) | (0.26 - 0.79) | (0.78 - 1.37) | (0.42 - 1.25) | (0.75 - 1.54) | (0.16 - 1.00) | (0.59 - 1.31) | (0.35 - 0.87) | (0.85 - 1.64) | (0.24 - 0.98) | (0.79 - 1.79) |
| Constant | 0.39*** | 0.29*** | 0.12*** | 0.10*** | 0.31*** | 0.26*** | 0.15*** | 0.14*** | 0.36*** | 0.24*** | 0.08*** | 0.07*** |
|  | (0.25 - 0.60) | (0.21 - 0.41) | (0.06 - 0.21) | (0.07 - 0.15) | (0.16 - 0.61) | (0.16 - 0.43) | (0.06 - 0.36) | (0.08 - 0.24) | (0.20 - 0.66) | (0.15 - 0.39) | (0.03 - 0.19) | (0.04 - 0.12) |
| Observations | 5,986 | 5,982 | 5,999 | 5,999 | 2,973 | 2,971 | 2,976 | 2,976 | 2,970 | 2,968 | 2,980 | 2,980 |
| Pseudo R-squared | 0.0872 | 0.0334 | 0.143 | 0.0554 | 0.0921 | 0.0332 | 0.141 | 0.0559 | 0.0923 | 0.0321 | 0.154 | 0.0669 |
| Legend: 95% confidence level in parentheses. Significance level *** p<0.01, ** p<0.05, * p<0.1 | | | | | | | | | | | | |

Table A.4: Information criteria for latent class models selection

| Number of latent class | Log-likelihood (ll) | Akaike's Information Criterion (AIC) | Bayesian Information Criterion (BIC) |  |
| --- | --- | --- | --- | --- |
| All waves |  |  |  |  |
| Model with one class | -9399.47 | 18804.95 | 18825.06 |  |
| Model with two classes | -7699.38 | 15412.76 | 15459.68 | better fit |
| Model with three classes | -7699.3782 | 15414.756 | 15468.380 |  |
| Model with four classes | -7699.3782 | 15418.756 | 15485.787 |  |
| Wave 1 |  |  |  |  |
| Model with one class | -1622.904 | 3251.808 | 3266.547 |  |
| Model with two classes | -1323.551 | 2661.102 | 2695.491 | better fit |
| Wave 2 |  |  |  |  |
| Model with one class | -1586.198 | 3178.396 | 3193.125 |  |
| Model with two classes | -1305.653 | 2625.306 | 2659.674 | better fit |
| Wave 3 |  |  |  |  |
| Model with one class | -1492.266 | 2990.532 | 3005.27 |  |
| Model with two classes | -1239.882 | 2493.765 | 2528.154 | better fit |
| Wave 4 |  |  |  |  |
| Model with one class | -1516.388 | 3038.776 | 3053.509 |  |
| Model with two classes | -1243.713 | 2501.427 | 2535.802 | better fit |
| Wave 5 |  |  |  |  |
| Model with one class | -1539.768 | 3085.536 | 3100.269 |  |
| Model with two classes | -1233.272 | 2480.543 | 2514.919 | better fit |
| Wave 6 |  |  |  |  |
| Model with one class | -1626.45 | 3258.898 | 3273.63 |  |
| Model with two classes | -1325.59 | 2665.186 | 2699.561 | better fit |

| Table A.5: Description of latent classes (three-class model) for the total sample | | | |
| --- | --- | --- | --- |
|  | All waves | | |
|  | Class 1 | Class 2 | Class 3 |
| Total sample |  | |  |
| Proportion of respondents in each class (%) | 68.9 | 17.1 | 14 |
| Proportion  of respondents that | | | |
| Felt depressed (%) | 0.002 | 87.4 | 38.1 |
| Felt lonely (%) | 5.6 | 75.9 | 39.5 |
| Felt anxious (%) | 3.6 | 76.2 | 45.6 |
| Number of respondents | 6,021 | |  |

Table A.6: Associations of class membership (three classes) with alcohol and cannabis use during the pandemic (Adjusted odds ratios)

|  | Cannabis use past week | Heavy episodic drinking | Increase in cannabis use | Increase in alcohol use |
| --- | --- | --- | --- | --- |
| Total sample |  |  |  |  |
| Three latent classes (1=no/low symptoms, 2=moderate-symptoms, and 3=High-symptoms class) | 1.57*** | 1.36*** | 2.03*** | 1.62*** |
|  | (1.43 - 1.72) | (1.26 - 1.47) | (1.80 - 2.29) | (1.50 - 1.76) |
| Subsample of women | |  |  |  |
| Three latent classes (1=no/low symptoms, 2=moderate-symptoms, and 3=High-symptoms class) | 1.48*** | 1.36*** | 1.85*** | 1.50*** |
|  | (1.29 - 1.69) | (1.22 - 1.51) | (1.54 - 2.21) | (1.34 - 1.68) |
| Subsample of men | |  |  |  |
| Three latent classes (1=no/low symptoms, 2=moderate-symptoms, and 3=High-symptoms class) | 1.66*** | 1.38*** | 2.18*** | 1.77*** |
|  | (1.46 - 1.88) | (1.24 - 1.54) | (1.86 - 2.55) | (1.58 - 1.98) |
| Legend: 95% confidence level in parentheses. Significance level *** p<0.01, ** p<0.05. | | | | |
| Note: Odds ratios adjusted for sex, age, marital status, education, ethnicity, living area, household income, the presence of children, and other people in the household. | | | | |

Table A.7: Description of latent classes for the total sample, the women subsample, and the men subsample across waves

|  | All waves | | Wave 1 | | Wave 2 | | Wave 3 | | Wave 4 | | Wave 5 | | Wave 6 | | |
| --- | --- | --- | --- | --- | --- | --- | --- | --- | --- | --- | --- | --- | --- | --- | --- |
|  | Class 1 | Class 2 | Class 1 | Class 2 | Class 1 | Class 2 | Class 1 | Class 2 | Class 1 | Class 2 | Class 1 | Class 2 | Class 1 | Class 2 | |
| Total sample |  | |  | |  | |  | |  | |  | |  |  | |
| Proportion of respondents in each class (%) | 75.6 | 24.4 | 73.9 | 26.1 | 76.2 | 23.8 | 76.3 | 23.7 | 76.0 | 24.0 | 77.1 | 22.9 | 74.1 | 25.9 | |
| Proportion  of respondents that | | | | | | | | | | | | | | |  |
| Felt depressed (%) | 1.5 | 78.5 | 0.4 | 77.0 | 0.9 | 85.8 | 1.5 | 72.9 | 1.1 | 74.5 | 3.0 | 82.7 | 2.1 | 77.9 | |
| Felt lonely (%) | 7.0 | 69.9 | 8.7 | 64.3 | 8.7 | 71.4 | 6.0 | 69.3 | 7.9 | 70.9 | 5.0 | 71.0 | 5.9 | 73.2 | |
| Felt anxious (%) | 5.7 | 71.8 | 6.2 | 80.2 | 7.0 | 67.6 | 4.9 | 66.5 | 3.7 | 68.3 | 5.4 | 74.1 | 7.1 | 73.6 | |
| Number of respondents | 6,021 | | 1,005 | | 1,002 | | 1,005 | | 1,003 | | 1,003 | | 1,003 | | |
| Subsample of women | | | | | | | | | | | | | | |  |
| Proportion of respondents in each class (%) | 73.0 | 27.0 | 70.6 | 29.4 | 76.1 | 23.9 | 73.9 | 26.1 | 72.0 | 28.0 | 70.8 | 29.2 | 72.7 | 27.3 | |
| Proportion  of respondents that | | | | | | | | | | | | | | |  |
| Felt depressed (%) | 2.3 | 78.7 | 1.8 | 79.0 | 2.9 | 87.3 | 3.8 | 75.0 | 0.0 | 71.9 | 1.4 | 77.7 | 3.6 | 79.3 | |
| Felt lonely (%) | 8.2 | 69.7 | 9.7 | 69.5 | 9.7 | 75.0 | 6.8 | 69.5 | 9.8 | 60.6 | 6.0 | 65.2 | 6.8 | 77.2 | |
| Felt anxious (%) | 6.4 | 71.6 | 8.1 | 80.8 | 7.3 | 74.3 | 5.6 | 64.6 | 3.2 | 63.6 | 5.2 | 70.5 | 8.6 | 72.4 | |
| Number of respondents | 2,987 | | 498 | | 497 | | 499 | | 492 | | 498 | | 503 | | |
| Subsample of men | | | | | | | | | | | | | | |  |
| Proportion of respondents in each class (%) | 78.6 | 21.4 | 78.2 | 21.8 | 79.1 | 20.9 | 79.5 | 20.5 | 79.5 | 20.5 | 82.5 | 17.5 | 75.9 | 24.1 | |
| Proportion of respondents that | | | | | | | | | | | | | | |  |
| Felt depressed (%) | 0.7 | 78.3 | 0.0 | 74.5 | 0.0 | 89.3 | 0.0 | 71.1 | 1.4 | 75.3 | 3.9 | 88.6 | 0.9 | 75.5 | |
| Felt lonely (%) | 5.9 | 70.2 | 7.7 | 59.7 | 8.1 | 69.6 | 5.2 | 71.2 | 5.9 | 82.5 | 4.1 | 79.7 | 5.2 | 67.8 | |
| Felt anxious (%) | 5.1 | 72.0 | 4.3 | 81.7 | 7.2 | 61.9 | 4.5 | 71.2 | 4.0 | 72.2 | 5.1 | 78.2 | 5.7 | 75.6 | |
| Number of respondents | 2,987 | | 504 | | 492 | | 501 | | 501 | | 497 | | 492 | | |

Table A.8: Factors associated with high-symptoms class membership with factor*wave interaction variables (Adjusted Odds Ratios) – pooled sample.

|  | Odds Ratio | | | [95% Confidence Interval] | | | | |
| --- | --- | --- | --- | --- | --- | --- | --- | --- |
| Wave |  | | |  | | |  | |
| 1 | 0.246** | | | 0.075 | | | 0.805 | |
| 2 | 0.755 | | | 0.251 | | | 2.272 | |
| 3 | 0.541 | | | 0.177 | | | 1.650 | |
| 4 | 0.699 | | | 0.228 | | | 2.148 | |
| 5 | 0.424 | | | 0.132 | | | 1.359 | |
| 6 (ref) |  | | |  | | |  | |
| Men (ref) |  | | |  | | |  | |
| Women | 1.166 | | | 0.862 | | | 1.579 | |
| wave#Women | | |  | | |  | |  |
| 1#1 | 1.355 | | | 0.879 | | | 2.090 | |
| 2#1 | 1.039 | | | 0.669 | | | 1.614 | |
| 3#1 | 1.261 | | | 0.815 | | | 1.952 | |
| 4#1 | 1.008 | | | 0.652 | | | 1.558 | |
| 5#1 | 1.268 | | | 0.814 | | | 1.976 | |
| Worry about contracting COVID-19 | 2.001*** | | | 1.320 | | | 3.033 | |
| Worry about contracting COVID-19#wave |  | | |  | | |  | |
| 1#1 | 1.755 | | | 0.940 | | | 3.280 | |
| 1#2 | 1.210 | | | 0.681 | | | 2.150 | |
| 1#3 | 0.858 | | | 0.488 | | | 1.508 | |
| 1#4 | 1.248 | | | 0.697 | | | 2.233 | |
| 1#5 | 1.322 | | | 0.718 | | | 2.434 | |
| Living with others | 1.154 | | | 0.711 | | | 1.873 | |
| Living with others #wave |  | | | |  |  |  |  |
| 1#1 | 0.931 | | | 0.463 | | | 1.873 | |
| 1#2 | 0.822 | | | 0.408 | | | 1.658 | |
| 1#3 | 1.346 | | | 0.678 | | | 2.672 | |
| 1#4 | 0.716 | | | 0.359 | | | 1.427 | |
| 1#5 | 0.744 | | | 0.377 | | | 1.470 | |
| Presence of children | 0.871 | | | 0.582 | | | 1.304 | |
| Presence of children#wave |  | | | |  |  |  |  |
| 1#1 | 1.342 | | | 0.765 | | | 2.354 | |
| 1#2 | 1.336 | | | 0.763 | | | 2.340 | |
| 1#3 | 0.853 | | | 0.474 | | | 1.535 | |
| 1#4 | 1.460 | | | 0.825 | | | 2.585 | |
| 1#5 | 2.199*** | | | 1.225 | | | 3.947 | |
| Income less than $40,000 (ref) |  | | | |  |  |  |  |
| Income of $40,000-$79,999 | 0.644 | | | 0.386 | | | 1.073 | |
| Income of $40,000-$79,999#wave |  | | | |  |  |  |  |
| 1#1 | 1.141 | | | 0.557 | | | 2.341 | |
| 1#2 | 1.123 | | | 0.551 | | | 2.290 | |
| 1#3 | 1.073 | | | 0.528 | | | 2.180 | |
| 1#4 | 1.514 | | | 0.736 | | | 3.112 | |
| 1#5 | 0.873 | | | 0.419 | | | 1.819 | |
| Income of $80,000-$119,999 | 0.567** | | | 0.328 | | | 0.981 | |
| Income of $80,000-$119,999#wave |  | | | |  |  |  |  |
| 1#1 | 1.509 | | | 0.703 | | | 3.241 | |
| 1#2 | 0.712 | | | 0.325 | | | 1.560 | |
| 1#3 | 0.895 | | | 0.415 | | | 1.933 | |
| 1#4 | 1.281 | | | 0.589 | | | 2.786 | |
| 1#5 | 0.803 | | | 0.369 | | | 1.747 | |
| Income $120,000+ | 0.373*** | | | 0.209 | | | 0.666 | |
| Income $120,000+#wave |  | | | |  |  |  |  |
| 1#1 | 1.646 | | | 0.735 | | | 3.688 | |
| 1#2 | 0.615 | | | 0.268 | | | 1.409 | |
| 1#3 | 0.784 | | | 0.343 | | | 1.791 | |
| 1#4 | 2.320** | | | 1.038 | | | 5.185 | |
| 1#5 | 1.474 | | | 0.655 | | | 3.316 | |
| Income missing | 0.573 | | | 0.321 | | | 1.025 | |
| Income missing #wave | | |  | | |  | |  |
| 1#1 | 1.086 | | | 0.480 | | | 2.461 | |
| 1#2 | 0.678 | | | 0.293 | | | 1.567 | |
| 1#3 | 0.822 | | | 0.361 | | | 1.871 | |
| 1#4 | 1.272 | | | 0.566 | | | 2.858 | |
| 1#5 | 0.857 | | | 0.375 | | | 1.958 | |
| College diploma (ref) | | |  | | |  | |  |
| High school | 0.830 | | | 0.463 | | | 1.489 | |
| High school#wave | | |  | | |  | |  |
| 1#1 | 1.336 | | | 0.590 | | | 3.025 | |
| 1#2 | 0.881 | | | 0.376 | | | 2.063 | |
| 1#3 | 1.038 | | | 0.461 | | | 2.339 | |
| 1#4 | 1.241 | | | 0.545 | | | 2.823 | |
| 1#5 | 2.026 | | | 0.874 | | | 4.695 | |
| Post-secondary | 1.207 | | | 0.737 | | | 1.977 | |
| Post-secondary#wave | | |  | | |  | |  |
| 1#1 | 0.942 | | | 0.466 | | | 1.903 | |
| 1#2 | 1.174 | | | 0.578 | | | 2.385 | |
| 1#3 | 0.907 | | | 0.439 | | | 1.873 | |
| 1#4 | 0.771 | | | 0.374 | | | 1.590 | |
| 1#5 | 1.689 | | | 0.806 | | | 3.538 | |
| University | 1.161 | | | 0.786 | | | 1.716 | |
| University#wave | | |  | | |  | |  |
| 1#1 | 0.684 | | | 0.389 | | | 1.202 | |
| 1#2 | 0.843 | | | 0.476 | | | 1.494 | |
| 1#3 | 0.790 | | | 0.439 | | | 1.421 | |
| 1#4 | 0.931 | | | 0.530 | | | 1.634 | |
| 1#5 | 0.935 | | | 0.518 | | | 1.688 | |
| White (ref.) | | |  | | |  | |  |
| Non-White | 0.918 | | | 0.649 | | | 1.300 | |
| Non-White#wave | | |  | | |  | |  |
| 1#1 | 1.175 | | | 0.722 | | | 1.913 | |
| 1#2 | 0.945 | | | 0.576 | | | 1.552 | |
| 1#3 | 1.275 | | | 0.772 | | | 2.106 | |
| 1#4 | 1.285 | | | 0.784 | | | 2.107 | |
| 1#5 | 1.070 | | | 0.647 | | | 1.770 | |
| Rural (ref.) |  | | |  | | |  | |
| Urban | 0.716 | | | 0.460 | | | 1.114 | |
| Urban#wave | | |  | | |  | |  |
| 1#1 | 1.988** | | | 1.037 | | | 3.811 | |
| 1#2 | 1.478 | | | 0.769 | | | 2.842 | |
| 1#3 | 1.685 | | | 0.864 | | | 3.288 | |
| 1#4 | 1.079 | | | 0.575 | | | 2.026 | |
| 1#5 | 2.787*** | | | 1.428 | | | 5.442 | |
| Suburban | 0.834 | | | 0.534 | | | 1.302 | |
| Suburban#wave | | |  | | |  | |  |
| 1#1 | 1.471 | | | 0.755 | | | 2.868 | |
| 1#2 | 1.221 | | | 0.626 | | | 2.382 | |
| 1#3 | 1.115 | | | 0.563 | | | 2.206 | |
| 1#4 | 0.916 | | | 0.480 | | | 1.747 | |
| 1#5 | 1.469 | | | 0.744 | | | 2.900 | |
| Single (ref.) | | |  | | |  | |  |
| Separated/ divorced/ widowed | 1.151 | | | 0.650 | | | 2.037 | |
| Separated/ divorced/ widowed#wave | |  |  |  |  |  |  |  |
| 1#1 | 1.127 | | | 0.503 | | | 2.526 | |
| 1#2 | 1.342 | | | 0.603 | | | 2.988 | |
| 1#3 | 1.660 | | | 0.735 | | | 3.746 | |
| 1#4 | 0.780 | | | 0.351 | | | 1.734 | |
| 1#5 | 0.491 | | | 0.211 | | | 1.140 | |
| Married | 0.784 | | | 0.496 | | | 1.239 | |
| Married#wave | | |  | | |  | |  |
| 1#1 | 1.148 | | | 0.603 | | | 2.183 | |
| 1#2 | 1.357 | | | 0.715 | | | 2.573 | |
| 1#3 | 0.928 | | | 0.482 | | | 1.787 | |
| 1#4 | 0.692 | | | 0.360 | | | 1.331 | |
| 1#5 | 0.548 | | | 0.288 | | | 1.044 | |
| Age 18-39 (ref.) | | |  | | |  | |  |
| Age 40-59 | 0.928 | | | 0.647 | | | 1.331 | |
| Age 40-59#wave | | |  | | |  | |  |
| 1#1 | 0.858 | | | 0.514 | | | 1.432 | |
| 1#2 | 0.987 | | | 0.589 | | | 1.655 | |
| 1#3 | 1.109 | | | 0.656 | | | 1.877 | |
| 1#4 | 0.808 | | | 0.478 | | | 1.366 | |
| 1#5 | 1.102 | | | 0.655 | | | 1.853 | |
| Age 60+ | 0.349*** | | | 0.218 | | | 0.558 | |
| Age 60+#wave | | |  | | |  | |  |
| 1#1 | 1.125 | | | 0.579 | | | 2.188 | |
| 1#2 | 0.747 | | | 0.368 | | | 1.515 | |
| 1#3 | 1.034 | | | 0.519 | | | 2.060 | |
| 1#4 | 1.367 | | | 0.698 | | | 2.678 | |
| 1#5 | 1.251 | | | 0.617 | | | 2.537 | |
| Constant | 0.511 | | | 0.233 | | | 1.119 | |

Legend: 95% confidence level in parentheses. Significance level *** p<0.01, ** p<0.05, * p<0.1

Table A.9: Adjusted odds ratios from models with time and latent class membership interaction with F-test results

|  | Cannabis use past week | Heavy Episodic Drinking | Increase in cannabis use | Increase in alcohol use | Cannabis use past week | Heavy Episodic Drinking | Increase in cannabis use | Increase in alcohol use |
| --- | --- | --- | --- | --- | --- | --- | --- | --- |
| High-symptom class | 2.34*** | 2.01*** | 3.88*** | 3.39*** | 2.90*** | 1.34 | 5.02*** | 1.48** |
|  | (1.62 - 3.38) | (1.46 - 2.76) | (2.45 - 6.16) | (2.42 - 4.75) | (1.92 - 4.39) | (0.96 - 1.86) | (2.75 - 9.15) | (1.01 - 2.16) |
| High-symptom class*wave 1 | 0.95 | 1.13 | 0.61 | 0.61** | 0.77 | 1.70** | 0.47 | 1.40 |
|  | (0.55 - 1.65) | (0.72 - 1.77) | (0.30 - 1.22) | (0.38 - 0.97) | (0.43 - 1.37) | (1.07 - 2.68) | (0.21 - 1.04) | (0.85 - 2.29) |
| High-symptom class*wave 2 | 0.82 | 0.75 | 1.03 | 0.78 | 0.66 | 1.13 | 0.79 | 1.80** |
|  | (0.47 - 1.42) | (0.48 - 1.19) | (0.51 - 2.04) | (0.49 - 1.25) | (0.37 - 1.18) | (0.71 - 1.81) | (0.36 - 1.74) | (1.10 - 2.96) |
| High-symptom class*wave 3 | 0.93 | 0.78 | 0.75 | 0.66 | 0.75 | 1.17 | 0.58 | 1.51 |
|  | (0.53 - 1.61) | (0.49 - 1.23) | (0.37 - 1.53) | (0.41 - 1.06) | (0.42 - 1.33) | (0.73 - 1.87) | (0.26 - 1.30) | (0.91 - 2.52) |
| High-symptom class*wave 4 | 0.95 | 0.83 | 0.89 | 0.78 | 0.77 | 1.25 | 0.69 | 1.79** |
|  | (0.55 - 1.65) | (0.53 - 1.31) | (0.44 - 1.81) | (0.48 - 1.26) | (0.43 - 1.37) | (0.78 - 1.99) | (0.31 - 1.55) | (1.07 - 2.98) |
| High-symptom class*wave 5 | 1.24 | 0.66 | 1.29 | 0.44*** | Ref | Ref | Ref | Ref |
|  | (0.71 - 2.15) | (0.42 - 1.05) | (0.61 - 2.74) | (0.26 - 0.72) |  |  |  |  |
| High-symptom class*wave 6 | Ref | Ref | Ref | Ref | 0.81 | 1.50 | 0.77 | 2.30*** |
|  |  |  |  |  | (0.46 - 1.40) | (0.95 - 2.37) | (0.36 - 1.64) | (1.39 - 3.79) |
| F-test chi2 statistics | 2.11 | 6.96 | 4.48 | 12.19 | 2.11 | 6.96 | 4.48 | 12.19 |
| F-test p_value | 0.833 | 0.223 | 0.483 | 0.0323 | 0.833 | 0.223 | 0.483 | 0.0323 |
|  |  |  |  |  |  |  |  |  |
|  | Cannabis use past week | Heavy Episodic Drinking | Increase in cannabis use | Increase in alcohol use | Cannabis use past week | Heavy Episodic Drinking | Increase in cannabis use | Increase in alcohol use |
| High-symptom class | 2.23*** | 1.67*** | 3.47*** | 2.64*** | 2.17*** | 1.56*** | 2.92*** | 2.24*** |
|  | (1.48 - 3.35) | (1.20 - 2.33) | (2.01 - 5.98) | (1.86 - 3.76) | (1.43 - 3.27) | (1.12 - 2.19) | (1.69 - 5.03) | (1.58 - 3.17) |
| High-symptom class*wave 1 | 1.00 | 1.36 | 0.68 | 0.78 | 1.03 | 1.45 | 0.81 | 0.92 |
|  | (0.56 - 1.78) | (0.86 - 2.15) | (0.32 - 1.44) | (0.49 - 1.26) | (0.57 - 1.84) | (0.91 - 2.30) | (0.38 - 1.71) | (0.58 - 1.48) |
| High-symptom class*wave 2 | 0.86 | 0.91 | 1.15 | 1.01 | 0.89 | 0.97 | 1.36 | 1.19 |
|  | (0.48 - 1.53) | (0.57 - 1.45) | (0.54 - 2.42) | (0.62 - 1.62) | (0.50 - 1.58) | (0.61 - 1.55) | (0.64 - 2.89) | (0.74 - 1.91) |
| High-symptom class*wave 3 | 0.97 | 0.94 | 0.84 | 0.85 | Ref | Ref | Ref | Ref |
|  | (0.55 - 1.73) | (0.59 - 1.50) | (0.39 - 1.81) | (0.52 - 1.38) |  |  |  |  |
| High-symptom class*wave 4 | Ref | Ref | Ref | Ref | 1.03 | 1.07 | 1.19 | 1.18 |
|  |  |  |  |  | (0.58 - 1.83) | (0.67 - 1.71) | (0.55 - 2.56) | (0.72 - 1.93) |
| High-symptom class*wave 5 | 1.30 | 0.80 | 1.45 | 0.56** | 1.34 | 0.85 | 1.72 | 0.66 |
|  | (0.73 - 2.32) | (0.50 - 1.28) | (0.64 - 3.25) | (0.34 - 0.93) | (0.75 - 2.39) | (0.53 - 1.37) | (0.77 - 3.85) | (0.40 - 1.10) |
| High-symptom class*wave 6 | 1.05 | 1.20 | 1.12 | 1.28 | 1.08 | 1.29 | 1.33 | 1.52 |
|  | (0.61 - 1.81) | (0.76 - 1.90) | (0.55 - 2.27) | (0.79 - 2.08) | (0.62 - 1.87) | (0.81 - 2.03) | (0.65 - 2.71) | (0.94 - 2.45) |
| F-test chi2 statistics | 2.11 | 6.96 | 4.48 | 12.19 | 2.11 | 6.96 | 4.48 | 12.19 |
| F-test p_value | 0.833 | 0.223 | 0.483 | 0.0323 | 0.833 | 0.223 | 0.483 | 0.0323 |
|  |  |  |  |  |  |  |  |  |
|  | Cannabis use past week | Heavy Episodic Drinking | Increase in cannabis use | Increase in alcohol use | Cannabis use past week | Heavy Episodic Drinking | Increase in cannabis use | Increase in alcohol use |
| High-symptom class | 1.92*** | 1.52** | 3.98*** | 2.66*** | 2.22*** | 2.27*** | 2.35*** | 2.07*** |
|  | (1.27 - 2.90) | (1.09 - 2.12) | (2.36 - 6.70) | (1.91 - 3.70) | (1.47 - 3.36) | (1.64 - 3.13) | (1.39 - 3.99) | (1.50 - 2.86) |
| High-symptom class*wave 1 | 1.16 | 1.49 | 0.59 | 0.78 | Ref | Ref | Ref | Ref |
|  | (0.65 - 2.07) | (0.94 - 2.37) | (0.28 - 1.23) | (0.49 - 1.23) |  |  |  |  |
| High-symptom class*wave 2 | Ref | Ref | Ref | Ref | 0.86 | 0.67 | 1.69 | 1.29 |
|  |  |  |  |  | (0.48 - 1.54) | (0.42 - 1.06) | (0.81 - 3.52) | (0.81 - 2.03) |
| High-symptom class*wave 3 | 1.13 | 1.03 | 0.73 | 0.84 | 0.97 | 0.69 | 1.24 | 1.08 |
|  | (0.63 - 2.02) | (0.64 - 1.65) | (0.35 - 1.55) | (0.52 - 1.35) | (0.54 - 1.74) | (0.43 - 1.09) | (0.58 - 2.63) | (0.68 - 1.73) |
| High-symptom class*wave 4 | 1.16 | 1.10 | 0.87 | 0.99 | 1.00 | 0.74 | 1.47 | 1.28 |
|  | (0.65 - 2.06) | (0.69 - 1.76) | (0.41 - 1.84) | (0.62 - 1.60) | (0.56 - 1.79) | (0.47 - 1.17) | (0.69 - 3.13) | (0.80 - 2.05) |
| High-symptom class*wave 5 | 1.51 | 0.88 | 1.26 | 0.56** | 1.31 | 0.59** | 2.13 | 0.71 |
|  | (0.85 - 2.70) | (0.55 - 1.41) | (0.57 - 2.77) | (0.34 - 0.91) | (0.73 - 2.33) | (0.37 - 0.93) | (0.97 - 4.71) | (0.44 - 1.17) |
| High-symptom class*wave 6 | 1.22 | 1.33 | 0.98 | 1.28 | 1.05 | 0.89 | 1.65 | 1.64** |
|  | (0.70 - 2.11) | (0.84 - 2.09) | (0.49 - 1.95) | (0.80 - 2.03) | (0.61 - 1.83) | (0.57 - 1.39) | (0.82 - 3.31) | (1.03 - 2.61) |
| F-test chi2 statistics | 2.11 | 6.96 | 4.48 | 12.19 | 2.11 | 6.96 | 4.48 | 12.19 |
| F-test p_value | 0.833 | 0.223 | 0.483 | 0.0323 | 0.833 | 0.223 | 0.483 | 0.0323 |
